# Neuronal Histone Methyltransferase EZH2 Regulates Neuronal Morphogenesis, Synaptic Plasticity, and Cognitive Behavior in Mice

**DOI:** 10.1007/s12264-023-01074-1

**Published:** 2023-06-16

**Authors:** Mei Zhang, Yong Zhang, Qian Xu, Joshua Crawford, Cheng Qian, Guo-Hua Wang, Jiang Qian, Xin-Zhong Dong, Mikhail V. Pletnikov, Chang-Mei Liu, Feng-Quan Zhou

**Affiliations:** 1 Department of Orthopaedic Surgery, Johns Hopkins University School of Medicine, Baltimore, 21205 USA; 2 The Solomon H. Snyder Department of Neuroscience, Johns Hopkins University School of Medicine, Baltimore, 21205 USA; 3 Department of Psychiatry and Behavioral Sciences, Johns Hopkins University School of Medicine, Baltimore, 21205 USA; 4 Department of Ophthalmology, Johns Hopkins University School of Medicine, Baltimore, 21205 USA; 5 School of Life Sciences, Division of Life Sciences and Medicine, University of Science and Technology of China, Hefei, 230026 China; 6 State Key Laboratory of Reproductive Biology, Institute of Zoology, Chinese Academy of Sciences, Beijing, 100190 China; 7 Sir Run Run Shaw Hospital, Zhejiang University School of Medicine, Hangzhou, 310016 China

**Keywords:** Neural development, Dendritic branching, Dendritic spine, Cognitive function, Epigenetics, Histone methylation, EZH2

## Abstract

The histone methyltransferase enhancer of zeste 2 polycomb repressive complex 2 subunit (EZH2)-mediated trimethylation of histone H3 lysine 27 (H3K27me3) regulates neural stem cell proliferation and fate specificity through silencing different gene sets in the central nervous system. Here, we explored the function of EZH2 in early post-mitotic neurons by generating a neuron-specific *Ezh2* conditional knockout mouse line. The results showed that a lack of neuronal EZH2 led to delayed neuronal migration, more complex dendritic arborization, and increased dendritic spine density. Transcriptome analysis revealed that neuronal EZH2-regulated genes are related to neuronal morphogenesis. In particular, the gene encoding p21-activated kinase 3 (*Pak3)* was identified as a target gene suppressed by EZH2 and H3K27me3, and expression of the dominant negative *Pak3* reversed *Ezh2* knockout-induced higher dendritic spine density. Finally, the lack of neuronal EZH2 resulted in impaired memory behaviors in adult mice. Our results demonstrated that neuronal EZH2 acts to control multiple steps of neuronal morphogenesis during development, and has long-lasting effects on cognitive function in adult mice.

**Supplementary Information:**

The online version contains supplementary material available at 10.1007/s12264-023-01074-1.

## Introduction

During the development of the central nervous system, post-mitotic neurons generated through neurogenesis undergo several steps of morphogenesis, including neuronal migration, neuronal polarization, axon growth/guidance, dendrite development, and synaptogenesis, to form functional neural circuits. Disruptions in these processes are believed to cause many neurodevelopmental disorders, such as autism spectrum disorder (ASD), schizophrenia, and intellectual disability. Previous studies have extensively investigated the roles of extracellular guidance cues, their receptors, and downstream signaling mediators, as well as cytoskeletal proteins, in the regulation of these processes. Epigenetic regulation independent of DNA sequence is an emerging key cellular mechanism for coordinated regulation of functionally relevant gene expression in neural development. Interestingly, perturbation of several histone methyltransferases, such as nuclear receptor binding SET domain protein 1 (NSD1) for histone H3 lysine 36 (H3K36) and mixed lineage leukemia 2 (MLL2) for histone H3 lysine 4 (H3K4), causes Sotos syndrome and Kabuki syndrome, respectively, both of which show intellectual disability [1, 2]. Mutations of histone-modifying proteins appear to be the causes of major neurodevelopmental disorders [3].

The polycomb repressive complex 2 (PRC2) is one of the two polycomb group proteins (PcG) that has histone methyltransferase activity and primarily trimethylates histone H3 on lysine 27 (H3K27me3) [4]. The mouse PRC2 has 4 subunits: SUZ12 polycomb repressive complex 2 subunit (SUZ12), embryonic ectoderm development (EED), EZH2, and retinoblastoma-binding protein p48 (RbAp48), among which EZH2 is the methyltransferase that trimethylates H3K27, and the histone demethylases ubiquitously transcribed tetratricopeptide repeat on chromosome X (UTX)/lysine demethylase 6A (KDM6A), and jumonji domain-containing protein D3 (JMJD3)/lysine demethylase 6B (KDM6B) remove methyl groups specifically from H3K27 [5]. Previous studies have identified EZH2 as one of the causative genes of Weaver syndrome, a developmental disorder characterized by macrocephaly, dysmorphic facial features, accelerated skeletal maturation, limb anomalies, developmental delay, and intellectual disability [6]. The genome-wide profiling of histone methylations has shown a significant difference in H3K27 methylation between olfactory cells (with neuronal traits) obtained from schizophrenia patients and those from control groups [7]. Moreover, both EZH2 and JMJD3 have been identified as susceptibility genes for ASD [8, 9]. These facts prompted us to study the function of EZH2 and H3K27 methylation in early neuronal development.

The main function of PRC2 and H3K27 methylation is to epigenetically maintain gene silencing [10, 11]. Previous studies in stem cell maintenance and differentiation have suggested that PRC2 functions to maintain cell identity at different stages of cell development. For instance, in stem cells, H3K27me3 represses the expression of differentiation-related genes to maintain the stem cell fate. When stem cells differentiate into specific cell types in response to extracellular signals, H3K27me3 instead represses stem cell genes and genes related to other cell types. Thus, H3K27me3 acts to repress different sets of genes in the same cell lineage at different developmental stages. EZH2 is highly expressed in dividing neural progenitors, and loss of EZH2 in neural progenitor cells disrupts neurogenesis in both embryonic and adult animals [12, 13]. When neural progenitors differentiate into post-mitotic neurons, the expression of EZH2 is down-regulated, but still maintained. Conditionally knocking out *Ezh2* in neural progenitors has been shown to regulate directional neuronal migration in the brain stem [14]. Similarly, knocking down EZH2 in radial glial cells leads to impaired cortical neuronal migration [15]. Interestingly, a recent study showed that activating endogenous EZH2 in embryonic stem cells using the clustered regularly interspaced short palindromic repeat (CRISPR)-mediated transcriptional activation approach promotes neuronal differentiation, likely by suppressing the expression of endoderm- and mesoderm-specific gene transcription [16]. Moreover, when combined with other transcription factors, such as *Pou3f2* and *Neurogenin 1*, EZH2 expression induces the direct reprogramming of fibroblasts to neurons. In summary, these studies highlight the roles of EZH2 in stem cells to control neuronal differentiation. However, the direct roles of EZH2 in post-mitotic neurons remain elusive.

To date, the specific *in vivo* roles of EZH2 in early post-mitotic neurons during development remain largely unknown. To address the question, we generated neuron-specific *Ezh2* conditional knockout mice using *Neurod6-Cre* mice [17], in which Cre recombinase is expressed specifically in early post-mitotic excitatory neurons of the developing brain. By using such mouse line, we set out to determine how neuronal EZH2 acts to control neural development and cognitive function in adult mice.

## Materials and Methods

### Animals

All experiments involving animals were performed in accordance with an animal protocol approved by the Institutional Animal Care and Use Committee of Johns Hopkins University. The *Neurod6-Cre* (*Nex-Cre*) mice were generously provided by William D. Snider. *Ezh2*^f/f^ (MMRRC_015499-UNC) mice were purchased from the Mutant Mouse Resource Research Center. *Ezh2*^f/f^ mice have been described [12, 18]. The Thy1-GFP M mice were generously provided by Richard Huganir [19]. The mice have been described. The day of vaginal plug detection was designated as embryonic day 0.5 (E0.5) and the day of birth as postnatal day 0 (P0).

### DNA Constructs and Antibodies

Plasmid pCAG-dsRed was from Addgene (Plasmid #11151). *Neurod1-Cre* was provided by Dr. Franck Polleux (Columbia University). *Pak3*^K297R^ was gifted by Dr. Rick Horwitz (University of Virginia). The PAK3 mutant was subcloned into the pCMVmyc vector.

The following primary antibodies were used: anti-EZH2 1:500 (BD Biosciences, San Jose, USA, Cat. No. 612666), anti-EZH2 1:1000 (Cell Signaling, Danvers, USA, Cat. No. #5246), anti-GAPDH 1:5000 (Sigma-Aldrich, St. Louis, USA, Cat. No. G8795), anti-GFP 1:1200 (Thermo Fisher Scientific, Waltham, USA, Cat. No. A10262), anti-TUJ1 1:1000 (Biolegend, San Diego, USA, Cat. No. 801201), anti-H3K27me3 1:2000 (Millipore, Burlington, USA, Cat. No. 07449), anti-H3 1:1000 (Cell Signaling, Cat. No. #4499), anti-dsRED 1:1000 (Clontech, Mountain View, USA, Cat. No. 632496), anti-Tbr1 1:1000 (Abcam, Waltham, USA, Cat. No. ab31940), anti-Ctip2 1:500 (Abcam, Cat. No. ab18465), anti-Foxp1 1:1000 (Abcam, Cat. No. ab16645), and anti-Cre 1:1000 (Millipore, Cat. No. MAB3120).

### Primary Neuronal Cultures and Treatments

All hippocampi and cortices were dissected in ice-cold Ca^2+^- and Mg^2+^-free Hank’s balanced salt solution (Gibco, Waltham, USA) and incubated with papain at 37°C for 15 min. Dissociated cells were plated on poly-D-lysine-coated 12 mm coverslips (Sigma). Cells were cultured on glass coverslips in Neurobasal medium (Gibco) containing 2% B27 supplement (Gibco) and 50 U/mL penicillin, 50 μg/mL streptomycin (Gibco), and 2 mmol/L GlutaMAX (Gibco), and grown for 5 or 18 days with media changes every other day. Transfection of the plasmids *pCAG-dsRed* and *Pak3*^K297R^ was applied at 7 days *in vitro* (DIV7) with 1 µg of DNA and 1.5 µL of Lipofectamine (Invitrogen, Carlsbad, USA), as described in the manufacturer’s manual (Invitrogen). After transfection, the culture medium was switched to the neuronal culture medium plus 2% fetal bovine serum (Gibco) with medium changes every other day. Neurons were fixed in 4% paraformaldehyde for 20 min and processed for immunocytochemistry.

### Golgi Staining

Freshly-dissected mouse brains were incubated in Golgi solutions A and B (FD Rapid GolgiStain Kit, FD NeuroTechnologies. Columbia, USA) for 10 days. After incubation, all brains were washed thoroughly with Solution C for 72 h at room temperature, and then the brains were blocked and embedded in optimal cutting temperature embedding medium (Tissue-Tek). Coronal sections (100 µm) through the somatosensory cortex and medial CA1 were cut on a Microm HM 550 cryostat (Thermo Scientific, Waltham, USA) and mounted on 3% gelatin-coated slides. Staining procedures were followed as described (FD NeuroTechnologies), and slides were dehydrated in ethanol and mounted with Permount (Fisher Scientific) for microscopy. Layer II–III neurons in the somatosensory cortex were included in our analyses.

### Immunohistochemistry and Fluorescence Intensity Quantification

Immunohistochemistry of cultured neurons and brains was performed and quantified using standard methods. In brief, the cultured cells were washed with PBS (Invitrogen) and then fixed in 4% paraformaldehyde (PFA). Neurons were blocked in 1% bovine serum albumin (BSA) in 0.2% phosphate-buffered saline-Tween (PBST), incubated with primary antibodies overnight at 4°C, and visualized with secondary antibodies.

For perfused brains, they were post-fixed in 4% PFA for 24 h at 4°C, followed by 15% and 30% (w/v) sucrose in PBS at 4°C until the brains sank. Brains were sectioned at 40–50 µm. The sections were incubated with primary antibodies overnight at 4°C and incubated with secondary antibodies for 1 h at room temperature.

All the images were taken on a Zeiss 510 confocal microscope (Zeiss, Oberkochen, Germany). Fluorescence intensity was quantified by ImageJ software (National Institute of Health, Bethesda, USA, available from http://rsb.info.nih.gov/ij). First, the background was subtracted from the fluorescence intensity of each image stack. Next, the average fluorescence per cell was calculated as their average mean values.

### Western Blot Analysis and Quantification

Tissue lysates were run on NuPAGE Novex 4%–12% Bis-Tris Protein Gels (Thermo Fisher Scientific). Western blotting was performed as described [20]. Novex gels were run at a constant 150 V to separate the proteins, which were transferred onto Immun-blot membranes (Bio-Rad, Hercules, USA) at a constant voltage (35 V for 3 h). The membranes were blocked using 5% milk prepared in Tris-buffered saline-Tween (TBS-T) [50 mmol/L Tris-HCl (pH 7.4), 150 mmol/L NaCl, 0.1% Tween-20] for 1 h at room temperature. The membranes were incubated with the primary antibodies overnight at 4°C. They were incubated with secondary antibodies conjugated with horseradish peroxidase (Thermo Fisher Scientific) for 1 h at room temperature. After washing with TBS-T, immunoreactivity signals were detected by enhanced chemiluminescence (Perkin-Elmer, Waltham, USA). Western blots were quantified by ImageJ software using the standard method.

### Morphological Analysis

All images were captured on a Zeiss 510 laser scanning confocal microscope. Dendritic processes, spine numbers, and the spine morphology of individual neurons of the somatosensory cortex and hippocampus were measured with ImageJ or Imaris.

Dendrites arising from the cell body were considered to be first-order segments until they bifurcated symmetrically into second-order segments; dendritic branches arising from the first-order segments were considered to be second-order segments until they bifurcated symmetrically into third-order segments. The following parameters for each reconstructed cortical neuron were analyzed: (1) total dendritic branch length, representing the summed lengths of dendritic segments; (2) total dendritic branch tip number, representing the total number of dendritic segments; and (3) average dendritic branch length, representing the individual dendritic branch lengths.

Spine density was calculated by counting the number of spines per measured length of the parent dendrite and expressed as the number of spines per 10-µm length of dendrite. The length of each dendritic segment used for spine densitometry was ~20 μm. For each neuron, spines in the second-order branches were quantified in P28–30 mice. Spine density in cultures was quantified from a 20-µm dendritic segment in the second-order branches of neurons.

### Dendrite Branching Analysis and Sholl Analysis

All GFP-positive image stacks from transfected cortical neurons were captured as described. Dendrite branching was calculated manually by tracing the dendrites. Sholl analysis was applied by drawing concentric circles centered on the cell soma using ImageJ. The starting radius was 5 µm and the interval between consecutive radii was 5 µm.

### Behavioral Studies

All behavioral experiments were performed on 3–4-month-old *Ezh2*^Δ/Δ^ mice and their *Ezh2*^flox/flox^ (control) littermates. Both male and female mice were included.

#### Y-Maze

The Y-maze test was used to assess working memory and short-term spatial recognition memory in mice. To test for working memory, spontaneous alternations were assessed. Mice were placed at the end of one of the three arms of the maze (San Diego Instruments, San Diego, USA) and allowed to explore the maze for 5 min. Videos were recorded and analyzed by Top Scan software (CleverSys Inc., Reston, USA). Spontaneous alternations were measured as the percentage of correct sequences of successive, non-repeated visits to all three arms. Five days later, the mice were tested again on the Y-maze for recognition memory. In trial 1, one of the three arms was blocked and the animals were allowed to explore the other two arms for 5 min; 12–15 min later, they were returned to the maze with no arms blocked and allowed to explore for 5 min. The time spent in each arm was recorded, with >33% of time spent in the previously blocked arm being identified as recognition memory. The time spent in the blocked arm was compared between the experimental and control mice.

#### Elevated Plus Maze

An elevated plus maze (EPM) test was used to test anxiety in mice. Mice were placed in the center of a T-shaped maze (San Diego Instruments) that had two open arms and two arms enclosed by walls. Mice were allowed in the EPM for 5 min. Videos of their movements were recorded and analyzed for the percentage of time spent in the open and closed arms by Top Scan software (CleverSys Inc.).

#### Novel Object Recognition

Object recognition memory was tested in the mice using a novel object recognition test. For 3 days prior to testing, mice were allowed to habituate to a custom-made 25 cm × 25 cm × 25 cm acrylic box for 10 min. On day 4, two identical objects were placed in the center of the box equidistant from each other and the walls and the mice were allowed to explore the objects for 10 min. One hour later, one of the two identical objects was replaced with a novel object. The mice were returned to the box and were allowed to explore the familiar and novel objects. The time spent sniffing the familiar and novel objects was automatically measured with Top Scan software (CleverSys Inc.).

#### Open Field Test

General locomotor activity was evaluated in activity chambers (San Diego Instruments) for 30 min. The time spent in the center and periphery of the arena, as well as the number of rears, were automatically assessed.

### *In Utero* Electroporation

All pregnant females were deeply anesthetized with avertin *via* intraperitoneal injection. Plasmids were mixed with fast green and microinjected into the lateral ventricle of embryos using a picospritzer (Parker, Cleveland, USA. Embryos were exposed at E15.5 and electroporated with 5 pulses of 35 V (50 ms on, 950 ms off at 1 Hz) through 5-mm tweezer electrodes connected to a square-wave electroporator (CUY21, π Protech, Boerne, USA).

### Chromatin Immunoprecipitation (ChIP) Assay

ChIP assays were performed with a commercial kit (Chip-IT kit, Active Motif, Carlsbad, USA). The cortices from P2 wild-type mice were minced and crosslinked in 1% formaldehyde (F8775, Sigma) for 15 min and the reaction was stopped by adding glycine (0.125 mol/L). Nuclei of the cells were precipitated, lysed, and sonicated on ice 20 times for 5 s (duty cycle 40%, microtip limit 4) (Vibra-Cell V 50, Sonics Materials, Newtown, USA) (an average fragment size of 400 bp). Part of the supernatant was saved as input. The antibody used was polyclonal anti-H3K27me3 (Millipore) and an unrelated rabbit IgG. Immunoprecipitates were mixed with protein G magnetic beads and incubated overnight at 4°C, then washed, and protein/DNA complexes were eluted with cross-links reversed by incubating in ChIP elution buffer plus proteinase K for 2 h at 65°C. DNA was purified using spin columns and analyzed in duplicate by quantitative real-time polymerase chain reaction (q-PCR). Primer sequences for q-PCR are listed in Table 1. Fold enrichment is expressed as the ratio of the H3K27me3 signal to the IgG signal.

**Table 1:** Primer sequences for q-PCR of H3K27me3 ChIP at PAK3 promoter region

Primer	Forward sequence	Reverse sequence
Region 1	AAATTTGAGTGAATACATGAGAGGG	TTTATCCCCTCCTCCTGGGAAGATAG
Region 2	CTTGAAACTGGTGTGTGTATAAAAAGCC	CCCTCTCATGTATTCACTCAAATTT
Region 3	AGTGCCATGATAACTTGTATTTGGA	GGCTTTTTATACACACACCAGTTTCAAG

### Quantitative Real-time PCR

Total RNA from control and knockout mice was extracted using Trizol reagent (Invitrogen). RNA samples were treated with DNase I (Invitrogen) and reverse transcription was performed using a transcriptor first strand cDNA synthesis kit (Roche, Basel, Switzerland). LightCycler 480 SYBR Green I master mix (Roche) was used for real-time PCR. The threshold cycle (Ct) was determined on the linear phase. Relative gene expression fold difference was calculated by 2^−Δnormalized Ct^. q-PCR primers are listed in Table 2.

**Table 2:** Primer sequences for q-PCR

Primer	Forward sequence	Reverse sequence
PAK1	GAAACACCAGCACTATGATTGGA	ATTCCCGTAAACTCCCCTGTG
PAK2	AACGGAGAGCTAGAAGACAAGC	TGGAACAGAAGGCAAAGGTTT
PAK3	TTGGATAACGAAGAAAAACCCCC	GGGCACATCTGTGAGCCATAG
HDAC1	AGTCTGTTACTACTACGACGGG	TGAGCAGCAAATTGTGAGTCAT
BDNF	TCATACTTCGGTTGCATGAAGG	AGACCTCTCGAACCTGCCC
Ube3C	CAAGGCCCAAAGTGTCTCTCG	ACTGCATTTTTCAATCTTCGCC
Adora2b	AGCTAGAGACGCAAGACGC	AGCTAGAGACGCAAGACGC
HoxA1	CCACCAGGGTTATGCTGGG	CGTGGAGAGGGGATAAGGAGTTA
Slc6a4	TATCCAATGGGTACTCCGCAG	CCGTTCCCCTTGGTGAATCT
PITX1	GCCTTCAAGGGAGGCATGAG	GATTCGCTGGCGGAGTTCTC
IGF2	GTGCTGCATCGCTGCTTAC	ACGTCCCTCTCGGACTTGG
EZH2	TGCCTCCTGAATGTACTCCAA	AGGGATGTAGGAAGCAGTCATAC

### Electrophysiology

Whole-cell electrophysiology and data analysis. Voltage-clamp whole-cell recordings were obtained from cultured neurons at room temperature (22–25°C). An external solution containing (in mmol/L) 150 NaCl, 3 KCl, 10 HEPES, 6 mannitol, 1.5 MgCl_2_, and 2.5 CaCl_2_, pH 7.4, was used for the recordings. Glass pipettes with a resistance of 20 MΩ were filled with an internal solution consisting of (in mmol/L) 110 cesium gluconate, 30 CsCl, 5 HEPES, 4 NaCl, 0.5 CaCl_2_, 2 MgCl_2_, 5 BAPTA, 2 Na_2_ATP, and 0.3 Na_2_GTP, pH 7.35. Neurons were held at a holding potential of −70 mV to record miniature excitatory synaptic currents (mEPSCs). To record mEPSCs, 1 µmol/L tetrodotoxin and 100 µmol/L picrotoxin were added to the external recording solution. The signal was filtered at 2 kHz and digitized at 20 kHz using an Axon Digidata 1440A analog-to-digital board (Molecular Devices, Sunnyvale, USA). Recordings were made from three independent cultures. Recordings with a pipette access resistance of 20 MΩ and with less than 20% change during the duration of recording were included. The mEPSC recordings were analyzed using Mini Analysis software (Synaposoft, Fort Lee, USA) with an amplitude threshold set at 6–8 pA. The frequency, amplitude, and decay were measured in each group.

### Genome-Wide RNA-Sequencing and Bioinformatics Analyses

Cortical neurons from either *Nestin-Cre*; *Ezh2*^f/f^ or *Neurod6-Cre*; *Ezh2*^f/f^ and their control littermates were isolated and cultured for 3 days. Then total RNA was isolated using an RNeasy Plus Kit (Qiagen, Venlo, Netherlands), RNA was quality controlled and quantified using an Agilent 2100 Bioanalyzer (Agilent, Santa Clara, USA). High-throughput sequencing was applied using the Illumina HiSeq 2000 platform (Illumina, San Diego, USA) at the Johns Hopkins University deep sequencing & microarray core.

The data analysis of RNA-seq used the following workflows. First, the transcript isoform level was analyzed by aligning the 54 base cDNA reads against the reference genome mm9 build using a Burrows-Wheeler transform-based short read aligner (BWA), and the aligned reads were visualized using the Integrated Genome Viewer. The read alignment files were imported into the Partek Genomics Suite (Partek® Genomics Suite^TM^, St. Louis, USA) and the RPKM (reads per kilobase of exon model per million mapped reads) counts for each of the 28,157 transcripts defined in the University of California at Santa Cruz (UCSC) refFlat annotation file were calculated. A stringent filtering criterion with an RPKM value of 1.0 was used to obtain transcripts. The RPKM values of filtered transcripts were log-transformed using log_2_(RPKM + offset) with an offset value of 1.0. Fold changes in transcript expression and *P-*values were computed using analysis of variances (ANOVA), and significantly altered transcripts were selected by applying a fold-change cutoff of 2. Alternatively, gene levels were analyzed by aligning the filtered reads to the mouse reference genome build mm9 using the ELAND (efficient local alignment of nucleotide data) algorithm (Anthony J Cox, Solexa Ltd. Saffron, Walden, UK). First, raw and RPKM-normalized counts were calculated for gene models (as defined in UCSC RefGene; http://genome.ucsc.edu/). Subsequently, we applied SAM (significance analysis of microarrays) to gene-level RPKM to identify RNAs with a fold-change greater than 2 and a false discovery rate of <25%. Briefly, the boundaries of exons were obtained from the RefGene database (http://genome.ucsc.edu/), and the numbers of mapped reads on each exon were calculated. To compare to human data sets, mouse gene names were converted to human homologs using the mouse genome informatics (MGI) annotation database (http://www.informatics.jax.org/homology.shtml). Gene Ontology (biological process) was assessed using Metascape (http://metascape.org/gp/index.html#/main/step1) web servers on substantially expressed genes, meaning a minimal log_2_(RPKM) of 1 among up-regulated genes upon EZH2 deletion, or a minimal log_2_(RPKM) of 1 among down-regulated genes in the control.

### Statistics

All plots were generated and statistically analyzed using GraphPad Prism version 7.0 (GraphPad, La Jolla, USA). Results are presented as the mean ± SEM. The sample size was not predetermined but the numbers of samples are consistent with previous publications. Two-tailed *t*-tests were used for the comparison of two data sets. Equal variances between groups and normal distributions of data were assumed but not formally tested. Molecular and biochemical analyses were performed using a minimum of three biological replicates per condition. Behavioral experiments require larger data sets due to increased variability. All statistical details of experiments, including the statistical tests used, the exact value of *n*, and the definition of *n*, can be found in the figure legends.

## Results

### Conditional Deletion of EZH2 in Post-mitotic Pyramidal Neurons in Mouse Brain

To confirm the expression of EZH2 in post-mitotic cortical neurons, we first applied immunostaining and detected the EZH2, SUZ12 (two major components of the PRC2 complex), and H3K27me3 all clearly in the nuclei of these neurons (Fig. S1A). To delete *Ezh2* specifically in post-mitotic neurons, we generated neuron-specific conditional* Ezh2* knockout mice by breeding *Ezh2* floxed mice (*Ezh2*^f/f^) with *Neurod6*-*Cre* (*Nex-Cre*) mice, in which the Cre recombinase is mainly expressed in early developing post-mitotic neurons starting from E11.5 [17]. It has been shown that *Neurod6-Cre*-mediated recombination is absent from proliferating neural precursors, interneurons, oligodendrocytes, and astrocytes [17]. Therefore, this Cre mouse line has been widely used to study gene functions in neurodevelopmental disorders specifically in post-mitotic neurons [21–25]. Indeed, immunostaining of Cre in P0 *Neurod6-Cre* mouse brain sections showed widespread Cre expression in the cortex and hippocampus (Fig. S1B).

Although both *Ezh2* and *Neurod6* genes are located in chromosome 6, we were able to obtain a few *Neurod6-Cre*/*Ezh2*^f/*+*^ mice, which were then bred with *Ezh2*^f/f^ mice again to obtain the conditional neuronal EZH2 knockout mice, *Neurod6*^*+/−*^*-Cre*/*Ezh2*^f/f^ (named *Ezh2*^Δ/Δ^ mice hereafter). Although the level of Neurod6 was reduced, it did not seem to affect the cognitive functions and morphogenesis of post-mitotic neurons based on previous studies [26, 27]. The Cre-mediated recombination was first verified by the reverse transcription-polymerase chain reaction (RT-PCR) in the *Neurod6-Cre*/*Ezh2*^*f/+*^ heterozygous mice. The results showed a mutant *Ezh2* band at 254 bp after *Neurod6-Cre*-mediated *Ezh2* deletion in one chromosome (Fig. S1C), while the remaining wild-type band was at 587 bp. The expression levels of EZH2 *in vivo* were evaluated by Western blot in homozygous *Ezh2*^Δ/Δ^ mice. The Western blot of postnatal day 0 (P0) mouse cortical lysate showed that the EZH2 level in *Ezh2*^Δ/Δ^ mice was dramatically decreased compared to the Cre*-*negative control littermates (*Ezh2*^f/f^*,* named control mice hereafter) (Fig. S1D). Because the whole cortical tissues were used for the Western blot samples, some of the remaining EZH2 protein was likely from glial cells or Cre-negative interneurons.

**Fig. 1:**
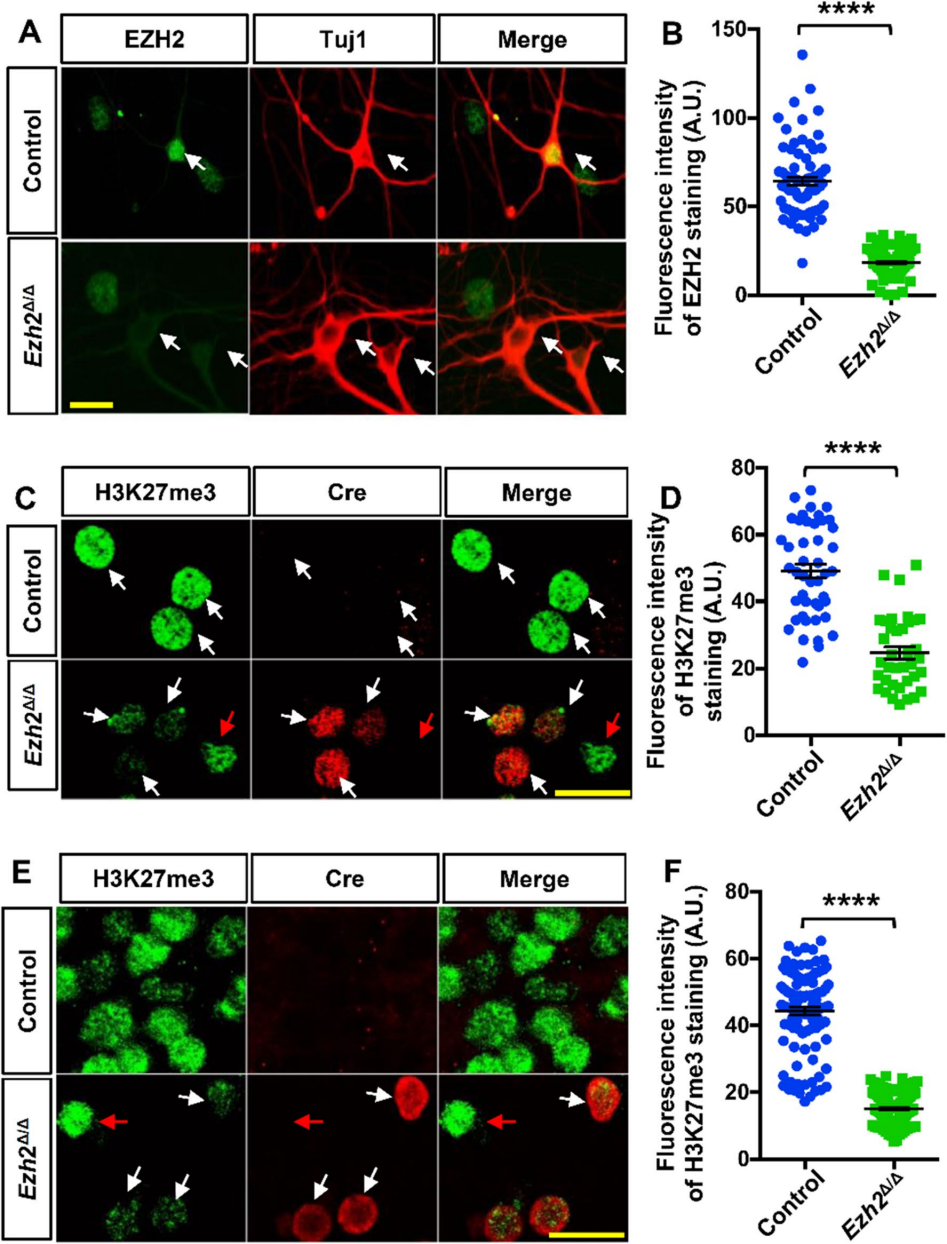
Deletion of H3K27 methyltransferase *Ezh2* in post-mitotic neurons.** A** Representative images showing the deletion of EZH2 in Cre-positive hippocampal neurons of *Ezh2*^Δ/Δ^ mice *in vitro*^*.*^ detected by immunostaining of EZH2. The white arrows indicate Tuj1-positive neurons. Scale bar, 20 µm. **B** Quantification of the fluorescence intensity of EZH2 staining shown in **A** (*n* = 74 and 91 for the control and *Ezh2*^Δ/Δ^ neurons, respectively, from three independent experiments; ****P < 0.0001, unpaired Student’s *t*-test). **C** Representative images showing the reduced level of H3K27me3 nuclear staining in Cre-positive hippocampal neurons *in vitro*. The white arrows in the upper panel indicate Cre-negative neurons from the control mice. The white arrows in the lower panel indicate the Cre-positive neurons from the *Ezh2*^Δ/Δ^ mice, while the red arrow indicates a Cre-negative neuron. Scale bar, 20 µm. **D** Quantification of the fluorescence intensity of the H3K27me3 staining shown in **C** (*n* = 45 and 37 for the control and *Ezh2*^Δ/Δ^ neurons, respectively, from three independent experiments; ****P < 0.0001, unpaired Student’s *t*-test). **E** Representative images showing the reduced level of H3K27me3 nuclear staining in cortical neurons from cortical slices of *Ezh2*^Δ/Δ^ mice *in vivo* by immunostaining of H3K27me3. The white arrows in the lower panel indicate the Cre-positive neurons in the *Ezh2*^Δ/Δ^ cortical slices, and the red arrow indicates a Cre-negative neuron. Scale bar, 20 µm. **F** Quantification of the fluorescence intensity of H3K27me3 staining shown in **E** (*n* = 103 and 111 neurons for the control and *Ezh2*^Δ/Δ^ neurons, respectively, from three independent experiments; ****P < 0.0001, unpaired Student’s *t*-test).

To directly show that EZH2 was deleted in post-mitotic neurons, we examined the expression level of EZH2 in cultured E18 hippocampal neurons from the control or *Ezh2*^Δ/Δ^ mice. EZH2 was clearly localized in the nuclei of wild-type neurons from the control mice, whereas it was drastically decreased in Cre-positive neurons from *Ezh2*^Δ/Δ^ mice (Fig. 1A, B). We next examined the fluorescence intensity of H3K27me3 immunostaining in cultured hippocampal neurons from *Ezh2*^Δ/Δ^ mice. The results showed that the levels of H3K27me3 in neuronal nuclei were significantly decreased in cultured Cre*-*positive neurons from *Ezh2*^Δ/Δ^ mice (Fig. 1C, D). We also examined the nuclear H3K27me3 levels in cortical slices and found a similar significant reduction in Cre-positive cortical neurons from *Ezh2*^Δ/Δ^ mice *in vivo* (Fig. 1E, F). Taken together, the results clearly demonstrated that EZH2 was successfully deleted in developing cortical and hippocampal neurons of *Ezh2*^Δ/Δ^ mice, and deleting EZH2 alone was sufficient to significantly reduce the H3K27me3 levels. *Ezh2*^Δ/Δ^ mice thus provided us with a useful model in which to study the specific functions of EZH2 and H3K27me3 in post-mitotic neurons.

### Neuronal EZH2 Regulates Migration of Post-mitotic Cortical Neurons *In Vivo*

To explore the neuronal functions of EZH2 during neural development, we analyzed the structure of the mouse cortex in both *Ezh2*^Δ/Δ^ mice and their littermate *Ezh2*^f/f^ controls at different developmental stages. The structure of the forebrain was analyzed by Nissl staining at P0, and no significant difference in brain structure was observed in *Ezh2*^Δ/Δ^ mutant mice (Fig. S2A). *Neurod6-Cre* is mainly expressed in post-mitotic neurons [17], and a small percentage has been identified in intermediate progenitors of the dorsal telencephalon [28] during early neuronal morphogenesis processes, such as neuronal migration. Thus, the Cre recombinase is likely efficiently expressed in later-born upper-layer cortical neurons at that particular time point. Indeed, when the cortical lamination was assessed at E15, a time point when earlier born deep layer cortical neurons are generated, the Tbr1-positive deep layer neurons appeared to be similar between the control and the *Ezh2*^Δ/Δ^ mice (Fig. S2B). The potential cause could be that the EZH2 protein might not be fully deleted in these early-born deep-layer post-mitotic neurons. To investigate the effects of neuronal EZH2 on the development of upper-layer cortical neurons, the mouse cortex was analyzed at P0 by Foxp1 and Ctip2 staining, which label layers II–IV and V–VI, respectively [29]. The distributions of labeled cells within 6 bins assigned across the apicobasal axis were quantified. We noted a milder but significant reduction of Foxp1-expressing neurons in *Ezh2*^Δ/Δ^ mice at the upper location (Bin 5) compared with that in control mice (Fig. S3A, B). In addition, the distributions of Ctip2-positive neurons were also significantly different between the *Ezh2*^Δ/Δ^ and control mice (Fig. S3A, C). The Ctip2-positive neurons in *Ezh2*^Δ/Δ^ mice were at a higher density in Bin 3 than those in wild-type mice. Together, these results suggested that the deletion of neuronal EZH2 by *Neurod6-Cre* significantly reduces the migration of upper cortical neurons.

To verify if EZH2 regulates the migration of post-mitotic neurons in a cell-autonomous manner and to visualize the morphologies of migrating neurons, we performed acute EZH2 deletion by *in utero* electroporation of *Neurod1-Cre* plasmid into E15 embryonic brains of *Ezh2*^f/f^ mice. Based on a previous study [30], the Neurod1 promoter only induced cDNA expression in intermediate progenitors and early postmitotic neurons in the subventricular zone (SVZ) and intermediate zone (IZ), but not Nestin-positive radial glial progenitors in the ventricular zone (VZ). The *pCAG-dsRed* plasmid was co-electroporated with *Neurod1-Cre* to label the transfected cells. The *Ezh2*^f/f^ embryos in the control group were only electroporated with the *pCAG-dsRed* plasmid (Fig. S3D). When the distribution of dsRed-positive cells was analyzed at P0, we found that acutely knocking out EZH2 in post-mitotic neurons significantly impaired neuronal migration. In contrast, the majority of neurons in the control mice migrated into the upper cortical plate. The data revealed that the deletion of EZH2 in post-mitotic neurons led to migration defects of upper-layer neurons at P0 (Fig. S3E). Because electroporation only transfected a small percentage of cells, the expression of Cre only generated some individual EZH2 knockout neurons in an otherwise controlled background. The results suggested that the EZH2 deletion-induced neuronal migration would be cell autonomous. Interestingly, when embryos were *in utero* electroporated at E15 and analyzed at P7, both control and *Ezh2*^Δ/Δ^ neurons (dsRed-positive) reached the upper layer similarly (Fig. S4). Thus, specific neuronal deletion of EZH2 only delayed but did not persistently impair the migration of cortical pyramidal neurons. Because this migration defect was similar to the finding of a previous study, in which EZH2 was deleted in neural stem cells [15], and the impact was not persistent, we decided not to pursue the underlying mechanisms further.

### Loss of Neuronal EZH2 Results in Increased Dendritic Complexity *In Vitro* and *In Vivo*

**Fig. 2:**
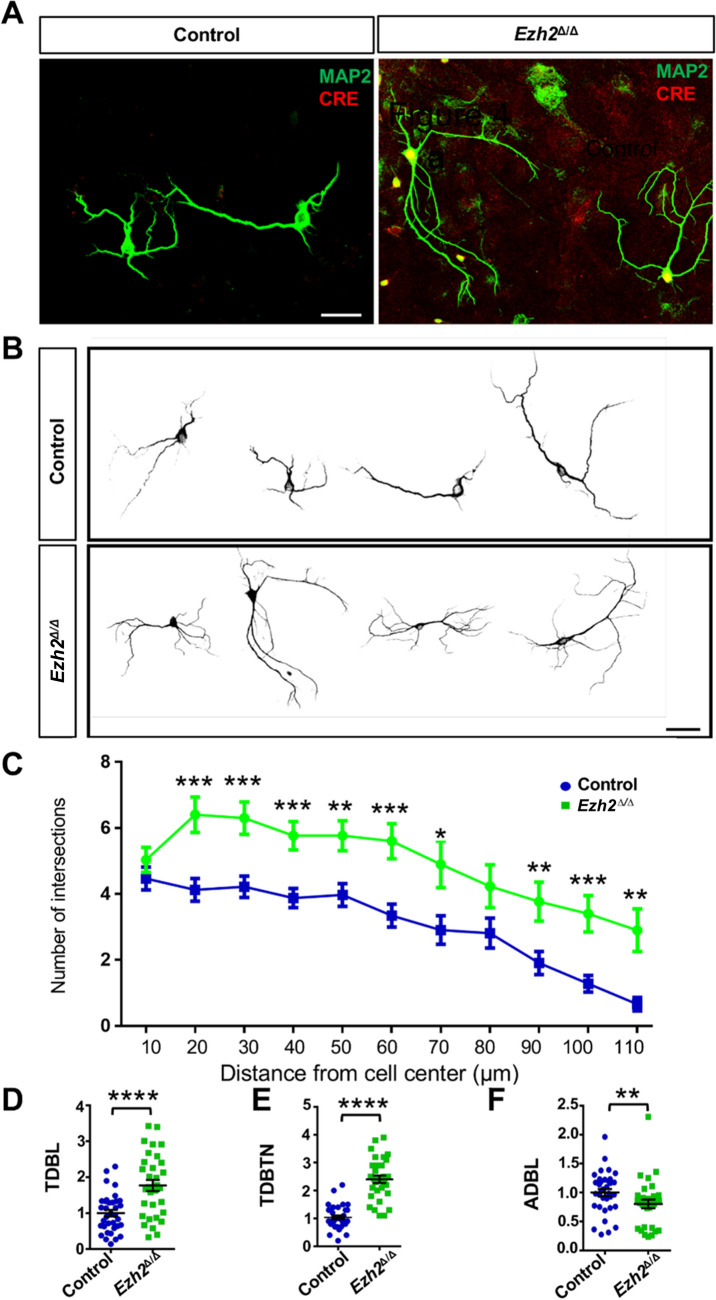
Loss of EZH2 increases dendritic branching *in vitro. A* Representative images of cultured DIV5 hippocampal neurons from control and *Ezh2*^Δ/Δ^ mice stained with Cre and MAP2 antibodies. Scale bar, 20 µm. **B** Representative images of several MAP2-stained hippocampal neurons from control and *Ezh2*^Δ/Δ^ mice. Note the increased dendritic branching of neurons from the *Ezh2*^Δ/Δ^ mice. Scale bar, 20 µm. **C** Sholl analysis of hippocampal neurons from control and *Ezh2*^Δ/Δ^ mice showing that, compared with that of the control mice, the neurons of the *Ezh2*^Δ/Δ^ mice have increased dendritic branching. (*P* = 0.2759 at 10 µm; *P* = 0. 0.0006 at 20 µm; *P* = 0.0006 at 30 µm; *P* = 0.0004 at 40 µm; *P* = 0. 0.0023 at 50 µm; *P* = 0.0006 at 60 µm; *P* = 0.0174 at 70 µm; *P* = 0.0757 at 80 µm; *P* = 0.0078 at 90 µm; *P* = 0.0007 at 100 µm; *P* = 0.0012 at 110 µm; *n* = 30 and 32 neurons for control and *Ezh2*^Δ/Δ^ mice, respectively, each from 3 to 4 mice, unpaired Student’s *t*-test). **D** Quantification of normalized total dendritic branch length (TDBL) of control and *Ezh2*^Δ/Δ^ neurons* (P* < 0.0001, *n* = 35 and 32 neurons from the control and the *Ezh2*^Δ/Δ^ mice, respectively, from 3 independent experiments, unpaired Student’s *t*-test). **E** Quantification of normalized total dendritic branch tip number (TDBTN) of control and *Ezh2*^Δ/Δ^ neurons (*P* < 0.0001, *n* = 35 and 32 neurons from the control and the *Ezh2*^Δ/Δ^ mice, respectively, from 3 independent experiments, unpaired Student’s *t*-test). **F** Quantification of normalized average dendritic branch length (ADBL) of control and *Ezh2*^Δ/Δ^ neurons (*P* = 0.0441, *n* = 35 and 32 neurons from the control and the *Ezh2*^Δ/Δ^ mice, respectively, from 3 independent experiments, unpaired Student’s *t*-test). In **C** and **D**, data are presented as the mean ± SEM; *P < 0.05, **P < 0.01, ***P < 0.001, ****P < 0.0001, compared to control if not designated.

To investigate the role of EZH2 in regulating neuronal morphogenesis, we isolated and cultured E18 hippocampal neurons from both control and *Ezh2*^Δ/Δ^ mice. At DIV5, the neurons were fixed and stained with both anti-Cre and anti-MAP2 antibodies to specifically label dendrites. To quantify dendritic morphology, we first evaluated dendrite branching using Sholl analysis, which measures the number of intersections of dendrites within each sphere plotted against radius increasing in 10-μm increments from the soma. Compared with the control neurons, *Ezh2*^Δ/Δ^ neurons showed more complex dendritic morphology (Fig. 2A, B). The Sholl analysis of dendritic arborization revealed markedly increased dendrite branching in *Ezh2*^Δ/Δ^ neurons compared to those of control neurons (Fig. 2C). Further, the morphological analysis revealed that the *Ezh2*^Δ/Δ^ neurons displayed significantly increased total dendritic branch length (TDBL) (Fig. 2D) and total dendritic branch tip number (TDBTN) (Fig. 2E). The average dendritic branch length (ADBL) was significantly reduced (Fig. 2F), likely due to the increased proportion of short branches. These results indicated that *Ezh2*^Δ/Δ^ hippocampal pyramidal neurons increased their dendritic growth and arborization by promoting the formation of short dendritic branches.

**Fig. 3:**
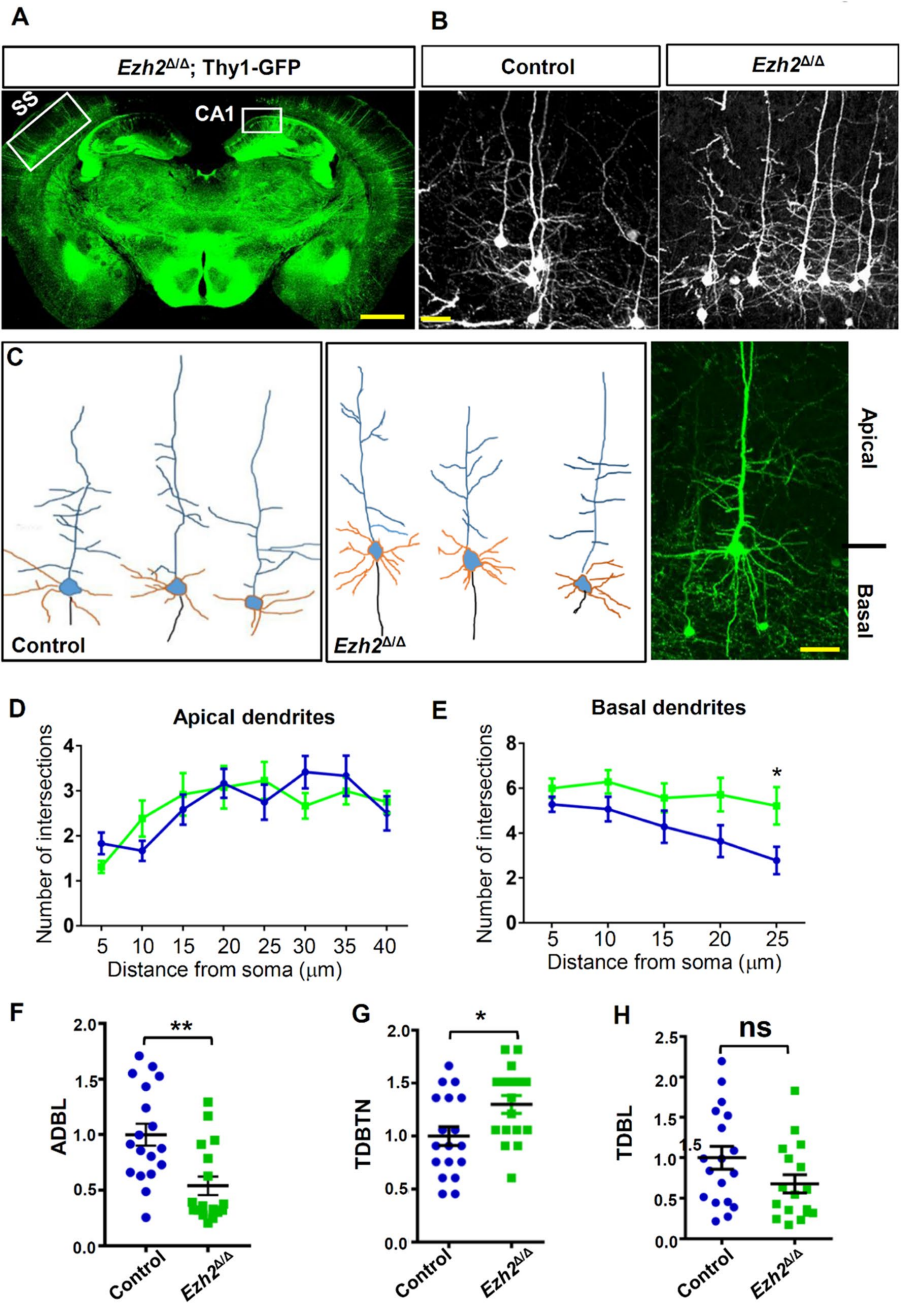
Lack of EZH2 increases the basal dendritic branching *in vivo.* **A** Representative image of an *Ezh2*^Δ/Δ^; Thy1-GFP mouse. GFP expression in the cortical and hippocampal regions. SS indicates the somatosensory cortex and CA1 indicates the CA1 region of the hippocampus. Scale bar, 1 mm. **B** Representative images of cortical neurons in *Ezh2 *^f/f^; Thy1-GFP and *Ezh2*^Δ/Δ^; Thy1-GFP mice. Scale bar, 20 µm. **C** Representative drawings of neurons in *Ezh2 *^f/f^; Thy1-GFP (left panel) and *Ezh2*^Δ/Δ^; Thy1-GFP (middle panel) mice. Orange. basal dendrites; blue. apical dendrites. Right panel. a representative image of a GFP-labeled cortical neuron. Scale bar, 20 µm. **D** Sholl analysis of the apical dendrites of cortical neurons showing no significant difference in dendritic trees between control and *Ezh2*^Δ/Δ^ cortical neurons (*n* = 14 neurons from 4 mice for each condition). **E** Sholl analysis of the basal dendrites of cortical neurons showing significantly increased complexity of *Ezh2*^Δ/Δ^ cortical neurons, compared with that of control mice (*P* = 0.03 at 25 μm, *n* = 14 neurons from 4 mice for each condition, unpaired Student’s *t*-test). **F** Quantification of normalized average dendritic branch length (ADBL) of control and *Ezh2*^Δ/Δ^ neurons *in vivo* (*P* = 0.0014, *n* = 18 and 17 neurons for the control and the *Ezh2*^Δ/Δ^ mice, respectively, from 4 mice from each condition, unpaired Student’s *t*-test). **G** Quantification of normalized total dendritic branch tip number (TDBTN) of control and *Ezh2*^Δ/Δ^ neurons *in vivo* (*P* = 0.0211, *n* = 18 and 17 neurons for the control and the *Ezh2*^Δ/Δ^ mice, respectively, from 4 mice from each condition, unpaired Student’s *t*-test). **H** Quantification of normalized total dendritic branch length (TDBL) of control and *Ezh2*^Δ/Δ^ neurons *in vivo* (*P* = 0.0879, *n* = 18 and 17 neurons for the control and the *Ezh2*^Δ/Δ^ mice, respectively, from 4 mice from each condition, unpaired Student’s *t*-test). In **D–H**, data are presented as the mean ± SEM; *P <0.05, **P <0.01, ns, not significantly different; compared to control if not designated.

To confirm that the dendritic morphology was altered in *Ezh2*^Δ/Δ^ mice *in vivo,* we generated *Ezh2*^Δ/Δ^;*Thy1-GFP-M* mice, in which pyramidal neurons in the adult cortex and hippocampus were labeled by Thy1-GFP. The control mice were *Ezh2*^f/f^;*Thy1-GFP-M* littermates. Because GFP-labeled neurons in the hippocampus were at a higher density, it was difficult to quantify the dendritic tree clearly. We thus prepared coronal brain sections of these mice at P28–30 and imaged the GFP-labeled layer IV–V neurons that were more sparsely labeled (Fig. 3A). Similar to cultured hippocampal neurons, *Ezh2*^Δ/Δ^ neurons exhibited more complex dendritic morphologies (Fig. 3B, C). Indeed, Sholl analyses revealed that *Ezh2*^Δ/Δ^ cortical neurons had more complex basal, but not apical, dendritic morphologies (Fig. 3D, E). Additional quantification showed that the basal dendrites of *Ezh2*^Δ/Δ^ neurons had significantly reduced ADBL and increased TDBTN, indicating increased dendrite branching with shorter branch lengths (Fig. 3F, G). These results were the same as those observed in cultured hippocampal neurons. However, unlike those *in vitro* results, the TDBL between the *Ezh2*^Δ/Δ^ and control neurons *in vivo* were similar (Fig. 3H). Overall, these results demonstrated that neuronal EZH2 functions to restrict dendritic development.

### Neuronal EZH2 Regulates Dendritic Spine Density and Synaptic Function

**Fig. 4:**
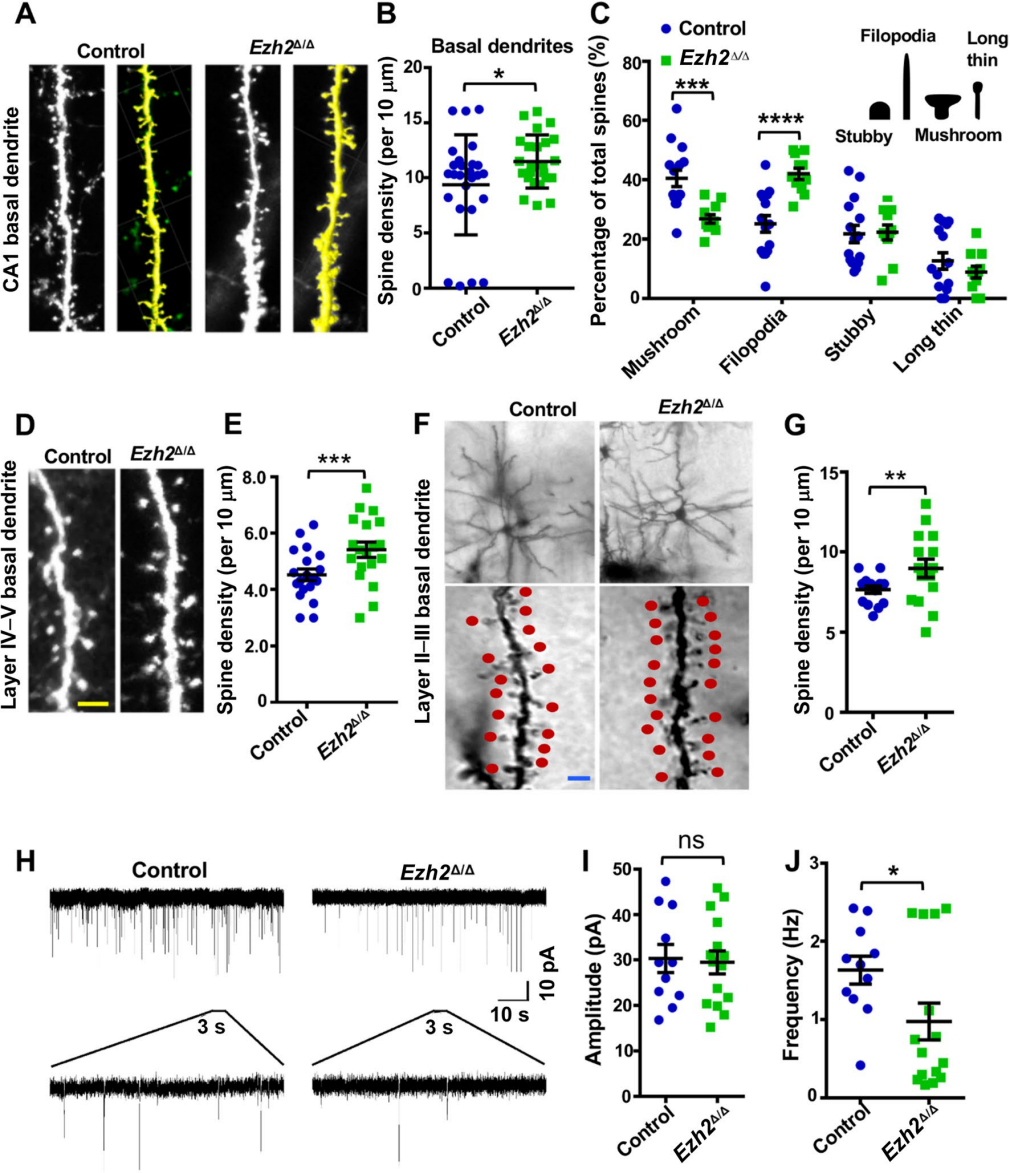
Dendritic spine density is increased in *Ezh2*^*∆/∆*^ mice. **A** Representative images of increased spine density in the basal region of hippocampal neurons from control and *Ezh2*^Δ/Δ^ mice. The yellow images are white images processed with the software Imaris. Scale bar, 5 µm. **B** Quantification of spine density in the basal region of hippocampal neurons from control and *Ezh2*^Δ/Δ^ mice (*P* = 0.0260, *n* = 28 and 24 neurons for the control and the *Ezh2*^Δ/Δ^ mice, respectively, from 4 mice for each condition, unpaired Student’s *t*-test). **C** Analysis of different spine types in the basal region of hippocampal neurons from control and *Ezh2*^Δ/Δ^ mice (*P* = 0.0003 for mushroom; *P* = 6.86624 × 10^−5^ for filopodia; *n* = 15 and 11 neurons for the control and the *Ezh2*^Δ/Δ^ mice, respectively, from 4 mice for each condition, unpaired Student’s *t*-test). The diagram shows the 4 different spine types. **D** Representative images of increased spine density in the basal region of layer IV–V neurons from *Ezh2 *^f/f^;Thy1-GFP and *Ezh2*^Δ/Δ^;Thy1-GFP mice. Scale bar, 5 µm. **E** Analysis of spine density in deep layer cortical neurons from control and *Ezh2*^Δ/Δ^ mice (*n* = 25 for control mice and *n* = 26 for *Ezh2*^Δ/Δ^ mice, *P* = 0.0002, unpaired Student’s *t*-test). **F** Representative images of increased spine density in the basal region of layer II–III neurons from *Ezh2 *^f/f^; and *Ezh2*^Δ/Δ^ mice labeled with Golgi staining. Scale bar, 5 µm. **G** Analysis of spine density in layer II–III cortical neurons from control and *Ezh2*^Δ/Δ^ mice (*n* = 25 for control mice and *n* = 26 for *Ezh2*^Δ/Δ^ mice*, P* = 0.0095, unpaired Student’s *t*-test). **H** Representative traces of mEPSCs of hippocampal neurons from control and *Ezh2*^Δ/Δ^ mice. **I, J** Quantification of average amplitude (**I**) and frequency (**J**) of mEPSCs of hippocampal neurons from control and *Ezh2*^Δ/Δ^ mice (amplitude: *P* = 0.8299, frequency: *P* = 0.0348; *n* = 11 and 15 neurons for the control and the *Ezh2*^Δ/Δ^ mice, respectively, from 4 independent experiments for each condition, unpaired Student’s *t*-test). Data are presented as the mean ± SEM. *P < 0.05, **P < 0.01, ***P < 0.001, ns, not significantly different; compared to control if not designated.

Synaptic remodeling in the hippocampus is a key process for learning and memory. Therefore, we examined dendritic spine development in the hippocampal and cortical pyramidal neurons of young adult control or *Ezh2*^Δ/Δ^ mice. We first examined P28–30 pyramidal neurons in the hippocampal CA1 region, and found a modest but significant increase in the spine density of the basal dendrites (Fig. 4A, B). Dendritic spines often show a myriad of morphologies, which are usually correlated with different synaptic properties. For instance, large mushroom-like spines usually form strong and persistent connections, whereas thin spines are more dynamic and have weaker connections. To better understand how EZH2 regulates dendritic spine development, we categorized the spine morphologies into 4 different groups: stubby spine, filopodia-like spine, mushroom spine, and long thin spine (Fig. 4C). Analysis of spine morphologies on the apical dendrites of CA1 neurons revealed that *Ezh2*^Δ/Δ^ neurons had a decreased percentage of mushroom spines and an increased percentage of filopodia-like spines. We next examined the dendritic spine density in layer IV–V cortical neurons from P28–30 *Ezh2*^Δ/Δ^;*Thy1-*GFP-M mice or *Ezh2*^f/f^;*Thy1-*GFP-M littermates. The spine density of basal dendrites was similarly increased in *Ezh2*^Δ/Δ^ mice (Fig. 4D, E). We also Golgi stained brain sections of P28–30 control or *Ezh2*^Δ/Δ^ mice. We found that the dendritic spine density of *Ezh2*^Δ/Δ^ layer II–III cortical neurons was significantly increased as well compared with that of the control neurons (Fig. 4F, G). These results indicated that EZH2 acted to restrict dendritic spine formation, and deletion of *Ezh2* led to increased dendritic spine density with less mature spines. It should be noted that the spine densities were different among the three observed neuronal types, including hippocampal CA1 neurons, and cortical layer IV–V and II–III neurons.

To investigate the functional consequences of increased dendritic spine density in *Ezh2*^Δ/Δ^ mice, we made whole-cell patch-clamp recordings of cultured hippocampal neurons *in vitro*. mEPSC analysis revealed a reduction in mEPSC frequency in *Ezh2*^Δ/Δ^ neurons, whereas no significant change was detected in the mEPSC amplitude (Fig. 4H–J). Changes in mEPSC amplitude often represent the density/conductance of postsynaptic receptors at individual synapses. Changes in mEPSC frequency are usually interpreted as changes in either the presynaptic release probability at existing sites or in the number of functional synaptic sites. As shown above, although *Ezh2*^Δ/Δ^ neurons exhibited more numerous synapses, the decreased number of mushroom spines and increased number of filopodia-like spines suggested a reduced functional synaptic connection. As a result, the reduction in mEPSC frequency in *Ezh2*^Δ/Δ^ mutant neurons was most likely due to the silencing of excitatory synapses.

### Transcriptome Analysis of Genes Regulated by Neuronal EZH2

**Fig. 5:**
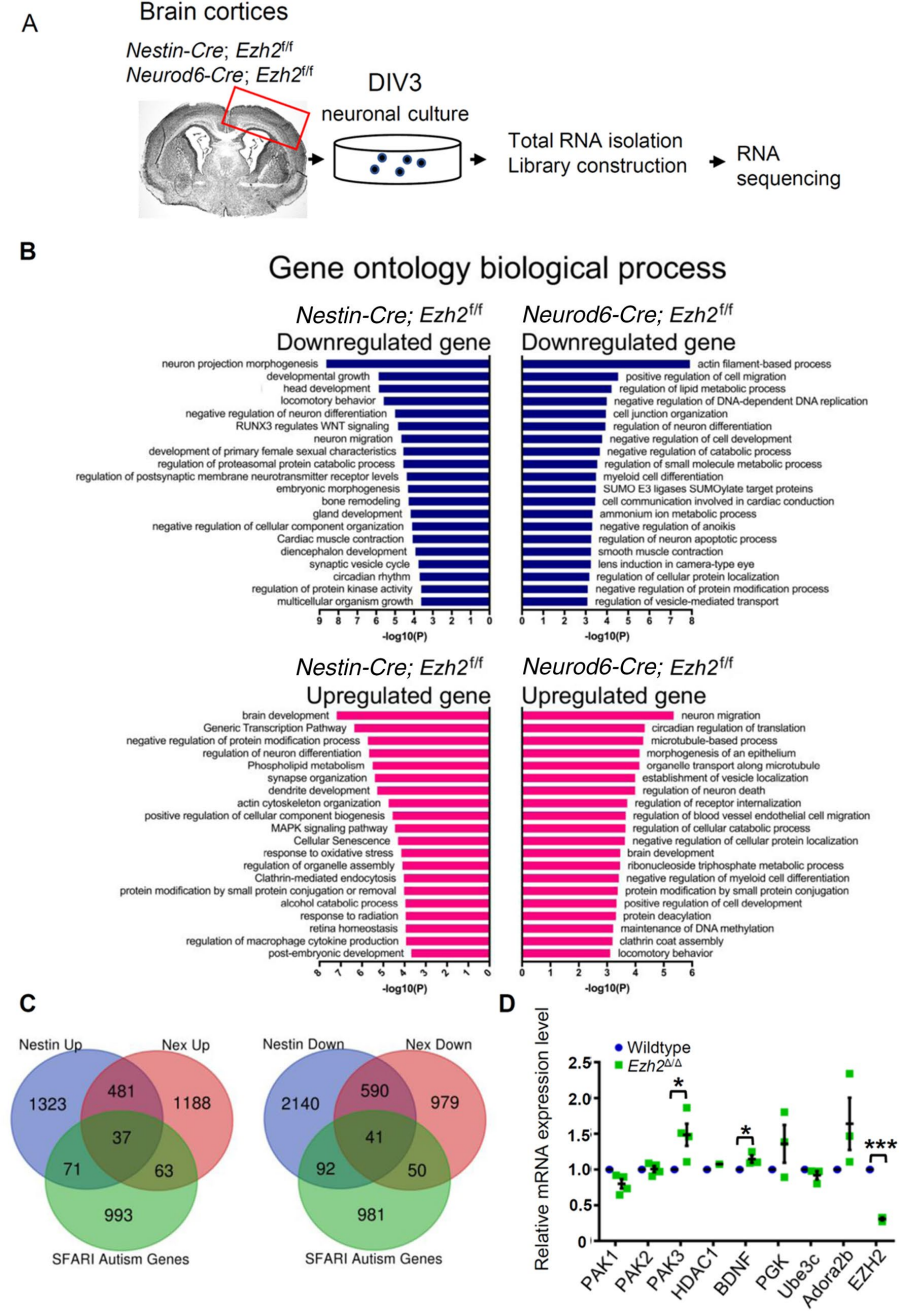
RNA-seq analysis of gene transcription in *Nestin-Cre*;*Ezh2*^f/f^ and *Ezh2*^*∆/∆*^ neurons. **A** Workflow of collecting excitatory neurons from control, *Nestin-Cre*;*Ezh2*^f/f^*,* and *Ezh2*^*∆/∆*^ mice for RNA-Seq analysis. **B** GO analysis of up-regulated and down-regulated differentially-expressed genes (DEGs) in *Nestin-Cre*;*Ezh2*^f/f^ and *Ezh2*^*∆/∆*^ mice, compared to that of the control mice. **C** Venn diagram representing the number of DEGs between SFARI autism genes and *Nestin-Cre*;*Ezh2 *^f/f^ or *Ezh2*^*∆/∆*^ mice. **D** Quantitative reverse transcription PCR (qPCR) analysis of selected DEGs in excitatory neurons in wild-type and *Ezh2*^*∆/∆*^ mice (*P* = 0.02 for PAK3, *P* = 0.02 for BDNF, *P* = 0.0007 for EZH2, *n* = 3 independent experiments, unpaired Student’s *t*-test). Data are presented as the mean ± SEM. *P <0.05, **P < 0.01, ***P < 0.001, compared to control if not designated.

To gain further insights into how EZH2 regulates gene expression in post-mitotic neurons, we compared the transcriptomes between control and *Ezh2*^Δ/Δ^ neurons, similar to a previously report in embryonic stem cells [11]. In addition to *Ezh2*^Δ/Δ^ mice, we also generated *Nestin-Cre/Ezh2*^f/f^ mice, in which *Ezh2* was knocked out in neural progenitors. To concentrate the excitatory neurons, hippocampal neurons from E18 *Ezh2*^f/f^, *Ezh2*^Δ/Δ^, or *Nestin-Cre*/*Ezh2*^f/f^ embryos were dissociated and cultured for 3 days in a serum-free neuronal culture medium favoring the survival of excitatory neurons. The neurons were collected to extract total RNAs, which were used for RNA-seq to identify differentially-expressed genes (DEGs) between control and *Ezh2* knockout neurons (Fig. 5A) (Tables S1 and S2). Our analysis revealed 1880 up- and 1783 down-regulated genes in the *Ezh2*^Δ/Δ^ neuronal samples compared with the control sample (>2-fold). In the sample from the *Nestin-Cre*/*Ezh2*^f/f^ neurons, there were 2061 up- and 3044 down-regulated genes (Fig. 5B, C). The DEGs identified through *Ezh2*^Δ/Δ^ mice and *Nestin-Cre/Ezh2*^f/f^ mice partially overlapped. Although the RNA-seq experiments were both performed using post-mitotic neurons, the results showed that knocking out *Ezh2* in neural progenitors or post-mitotic neurons resulted in different changes in gene transcription in neurons, confirming that EZH2 differentially regulates gene transcription in neural progenitors and post-mitotic neurons. To better understand the potential functions of these DEGs regulated by EZH2, we performed a Gene Ontology (GO) analysis of biological processes (Fig. 5B). The results showed that many DEGs induced by deleting neuronal EZH2 were related to biological processes involved in neural development, such as neuronal migration, positive regulation of cell migration, neuron projection morphogenesis, regulation of neuron differentiation, synapse organization, and dendrite development. Moreover, using quantitative real-time PCR (qPCR), we next examined a subset of genes that have been shown to be involved in the regulation of dendritic development or synaptic function. Several of these genes showed significant upregulation in *Ezh2*^Δ/Δ^ neurons, including *Pak3* and *Bdnf* (Fig. 5D). Together, these data suggested that neuronal EZH2 functions to regulate gene transcriptions related to neuronal morphogenesis.

We also performed network analyses of subgroups of enriched GO terms. The relationships among different GO terms were plotted and visualized using Metascape (http://metascape.org) and Cytoscape5 (Fig. S5). Moreover, by comparing DEGs induced by EZH2 knockout with the Simons Foundation Autism Research Initiative (SFARI) autism database (AutDB) (https://gene.sfari.org), we found that many DEGs overlapped with ASD risk genes (Fig. 5C). This result is consistent with recent studies, in which EZH2 was identified to be involved in the genetic etiology of autism [9] or an important downstream target of the gene encoding Chromodomain-Helicase-DNA-binding protein 8 (CHD8), a major ASD-associated gene [31].

### PAK3 Acts Downstream of EZH2 to Regulate Dendritic Spine Development

**Fig. 6:**
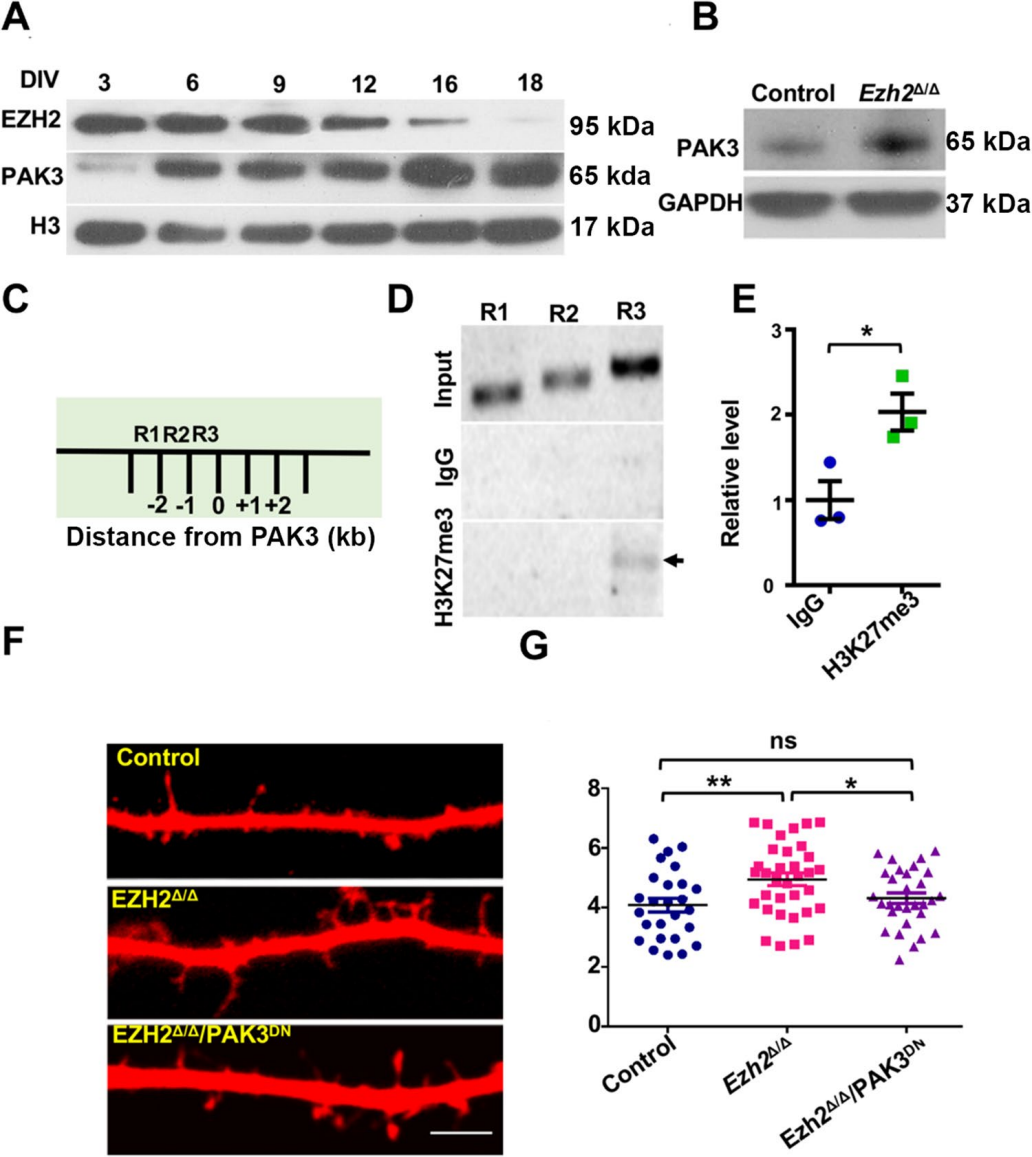
EZH2 regulates dendritic spine development by repressing the PAK3 gene. **A** Representative Western blot images showing the expression of EZH2 and PAK3 in hippocampal neurons after different periods in culture. **B** Representative Western blot images showing an increased level of PAK3 in neuronal lysates from *Ezh2*^*∆/∆*^ mice, compared to that from control mice. **C** Diagram showing the PAK3 promoter region, which is artificially separated into three regions, R1 to R3. **D** Representative PCR gel images showing H3K27me3 antibody-associated ChIP analysis using P7 mouse cortical tissue. The black arrow indicates the binding of the H3K27me3 antibody to the R3 region of the *PAK3* promoter. **E** qPCR analysis of the H3K27me3 ChIP assay shown in **D** (*P* = 0.029, *n* = 3 independent experiments, unpaired Student’s *t*-test). **F** Representative images of dendritic spines of cultured control neurons, *Ezh2*^*∆/∆*^ neurons, and *Ezh2*^*∆/∆*^ neurons expressing dominant negative PAK3 (PAK3^DN^). Note the increased filopodia-like spines on dendrites of *Ezh2*^*∆/∆*^ neurons. Scale bar, 5 μm. **G** Quantification of spine densities shown in **F** (*n* = 26, 31, and 30 neurons for the control, *Ezh2*^*∆/∆*^, and PAK3^DN^ neurons, respectively, from 4 independent experiments. *P* = 0.0079 between control and *Ezh2*^*∆/∆*^ neurons; *P* = 0.0293 between *Ezh2*^*∆/∆*^ and PAK3^DN^ neurons, unpaired Student’s *t*-test). Data are presented as the mean ± SEM. n.s., no significant difference, *P < 0.05, **P < 0.01, compared to control if not designated.

To provide evidence that neuronal EZH2 regulates dendritic development through its downstream targeted genes, we selected PAK3 because it is well known to regulate dendritic development and synaptogenesis, in a way similar to the phenotypes reported in *Ezh2*^Δ/Δ^ mice [32, 33]. In cultured hippocampal neurons, the protein levels of EZH2 decreased along with neuronal differentiation, whereas the levels of PAK3 increased (Fig. 6A). This result was consistent with the role of EZH2 in the regulation of H3K27me3, which functioned to repress gene expression. In support, Western blot analysis showed that the protein levels of PAK3 were significantly increased in *Ezh2*^Δ/Δ^ neurons (Fig. 6B). We performed ChIP-PCR experiments to determine if H3K27me3 directly interacted with the promoter region of *Pak3*. The genomic region of *Pak3* was divided into 3 overlapping fragments (R1–3, Fig. 6C), and specific primers were designed to amplify these 3 regions of the *Pak3* promoter. The result showed that H3K27me3 specifically interacted with the R3 region (from 2 kb to 1 kb upstream) but not the R1/2 regions in P7 mouse cortical tissue (Fig. 6D). In contrast, immunoprecipitation with IgG did not display any interactions with R1–3 regions. The qRT-PCR data showed that the binding of H3K27me3 with the R3 region was significantly higher than that with IgG in mouse cortical tissue (Fig. 6E). These results suggested that EZH2 represses *Pak3* expression through H3K27me3, which directly binds to the regulatory sequence upstream of *Pak3*.

To determine if PAK3 is indeed functionally involved in spine development downstream of EZH2, we tested if a dominant negative *Pak3* plasmid, *Pak3*^K297R^ [34], could revert the increased spine density observed in *Ezh2*^Δ/Δ^ neurons. E18 hippocampal neurons from the control or *Ezh2*^Δ/Δ^ mice were cultured. At DIV7, the control neurons were transfected with *dsRed*, and the *Ezh2*^Δ/Δ^ neurons were transfected with either *dsRed* or *dsRed* + *Pak3*^K297R^. At DIV18, the neurons were fixed and the dendritic spine densities were quantified. The results showed that the increased spine density in *Ezh2*^Δ/Δ^ neurons was reverted by *Pak3*^K297R^ to the same level as that of the control neurons (Fig. 6F, G). Altogether, these results demonstrated that EZH2 repressed the expression of PAK3, which regulated the spine density in hippocampal neurons. Importantly, in a previous study [34], overexpression of wild-type PAK3 increases the number of spines, as well as changing the morphology to long and thin dendritic protrusions. Such results are very similar to the results we obtained in the *Ezh2*^Δ/Δ^ neurons, confirming that PAK3 acts downstream of EZH2 to regulate dendritic spines. It should be noted that the spine density in cultured hippocampal neurons was much lower than that of *in vivo* samples, likely due to the much more complex microenvironment of the hippocampal tissue *in vivo*.

### Neuronal EZH2 Ablation During Development Has Prolonged Effects on Impairing Cognitive Behaviors in Adult Mice

**Fig. 7:**
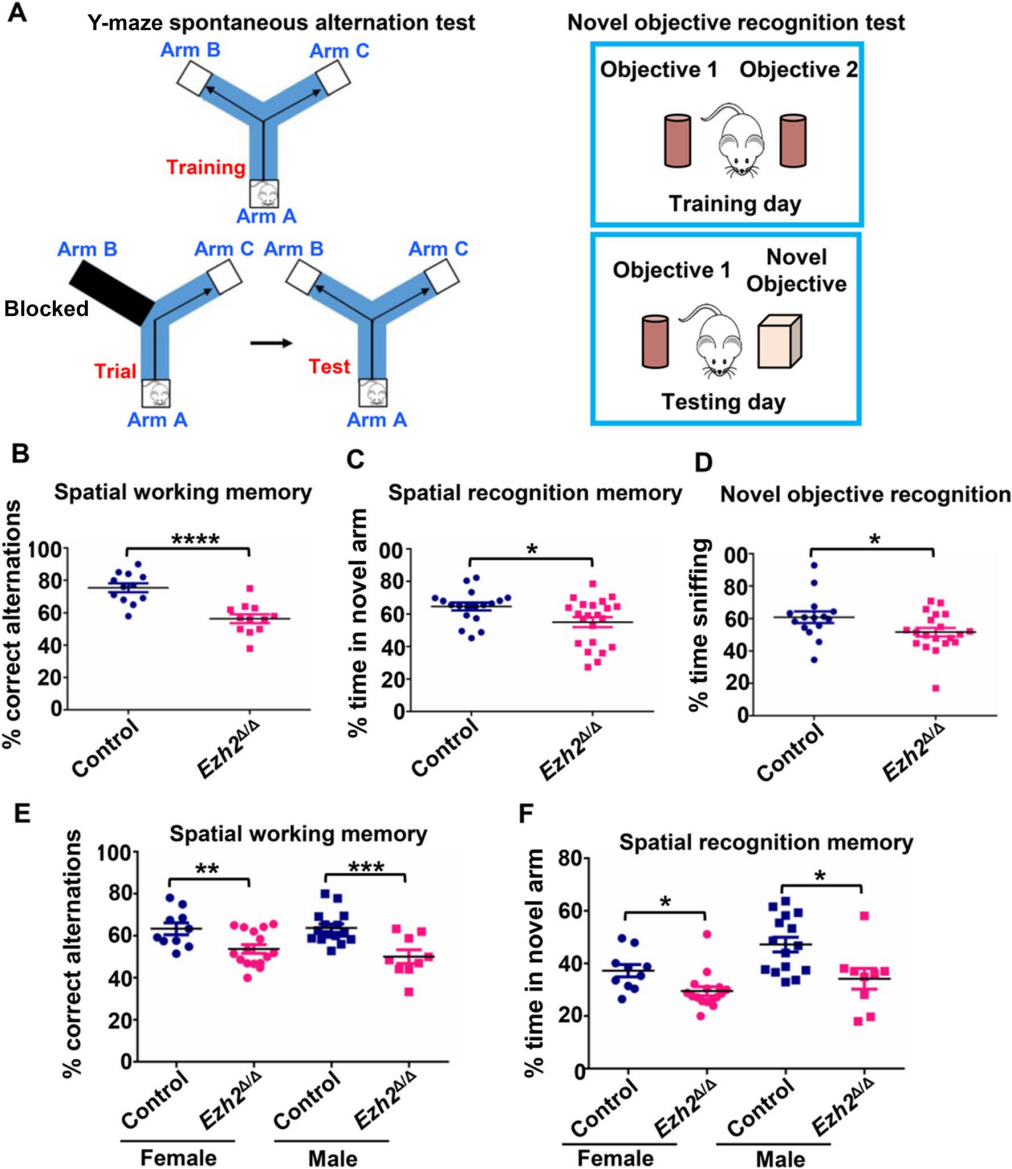
*Ezh2*^Δ/Δ^ mutant mice show deficient working and recognition memory. **A** Left: diagram of Y-maze spontaneous alternation test for examining spatial working memory and spatial recognition memory. Right: diagram of the novel object recognition test. **B** Quantification of the percentage of correct alternation between Y-maze arms. The result revealed that *Ezh2*^Δ/Δ^ mutant mice have impaired spatial working memory (*P* = 3.02523 × 10^−5^, *n* = 12 for either *Ezh2*^Δ/Δ^ or control mice, unpaired Student’s *t*-test). **C** Quantification of the percentage of time spent in the novel arm relative to the total duration of visits to the three arms during the test phase. Note that *Ezh2*^Δ/Δ^ mutant mice showed reduced recognition memory (*n* = 25 for control mice and *n* = 18 for *Ezh2*^Δ/Δ^ mice, *P* = 0.0174, unpaired Student’s *t*-test). **D** Quantification of novel object recognition memory in *Ezh2*^Δ/Δ^ mutant mice. Note that *Ezh2*^Δ/Δ^ mice spent a shorter percentage of time on the novel object (*n* = 15 for control mice and *n* = 20 for *Ezh2*^Δ/Δ^ mice, *P* = 0.0423, unpaired Student’s *t*-test). **E** Quantification of the percentage of alternation between Y maze arms. The result revealed that both male and female *Ezh2*^Δ/Δ^ mutant mice have impaired short-term working memory (female: *n* = 10 for control mice and *n* = 16 for *Ezh2*^Δ/Δ^ mice, *P* = 0.01; Male: *n* = 15 for control mice and *n* = 9 for *Ezh2*^Δ/Δ^ mice, *P* = 0.001, unpaired Student’s *t*-test). **F** Quantification of the percentage of time spent in the novel arms. The result revealed that both male and female *Ezh2*^Δ/Δ^ mice have impaired short-term working memory (female: *n* = 10 for control mice and *n* = 16 for *Ezh2*^Δ/Δ^ mice, *P* = 0.0265; Male: *n* = 15 for control mice and *n* = 9 for *Ezh2*^Δ/Δ^ mice, *P* = 0.0251, unpaired Student’s *t*-test). Data are presented as the mean ± SEM. n.s., no significant difference, *P < 0.05, **P < 0.01, ***P < 0.001, compared to control if not designated.

To investigate the potential effects of neuronal EZH2 ablation on the cognitive functions of adult mice, we performed a battery of behavioral tests using *Ezh2*^Δ/Δ^ and control (*Ezh2*^f/f^) littermate male and female mice. Spatial working memory and spatial recognition memory were evaluated using the Y-maze assay as previously described (Fig. 7A) [35]. To test for working memory, the spontaneous alternations test was applied. We found that *Ezh2*^Δ/Δ^ mutant mice showed a significant reduction in the percentage of correct alternations compared to control mice (Fig. 7B). Mice were also tested in the Y-maze for recognition memory. We found that *Ezh2*^Δ/Δ^ mice spent a shorter time in the novel arm (the arm that was first blocked) (Fig. 7C). These results indicate that *Ezh2*^Δ/Δ^ mice had reduced spatial working and recognition memories. We next applied the novel object recognition test (Fig. 7A, D). The results showed that *Ezh2*^Δ/Δ^ mutant mice spent significantly less time sniffing the novel object, indicating that novel object recognition memory was also impaired in these mice.

Using a different group of mice, we applied similar spatial working and recognition memory tests by separately analyzing the results from male or female mice. We found that both male and female *Ezh2*^Δ/Δ^ mice had significantly impaired spatial working and recognition memories (Fig. 7E, F). These cognitive deficits could not be explained by changes in anxiety or general activity as no group difference in the elevated plus maze test (Fig. S6A, B) or the open field test (Fig. S6C–E) was found. Collectively, these results illustrated that working and recognition memory was significantly impaired in the *Ezh2*^Δ/Δ^ mice, suggesting that neuronal EZH2 might be involved in neuronal development processes contributing to memory-related cognitive functions in adult mice.

## Discussion

The major function of EZH2 is to trimethylate H3K27 and repress gene expression, which has been mostly studied in dividing cells, such as stem cells and cancer cells. During different stages of cell development, including the maintenance of the stem cell states and distinct cell fate differentiation, EZH2-mediated H3K27me3 acts to repress distinct gene sets in different cell types to maintain their specific identities. For instance, in stem cells, H3K27me3 functions to maintain stemness by repressing genes that induce cell differentiation. However, after stem cells differentiate into specific cell types, H3K27me3 instead regulates genes related to cell differentiation and maturation. Disruption of such function often leads to failed cell differentiation and tumorigenesis [36]. In the nervous system, knocking out *Ezh2* in neural progenitors suppresses proper progenitor cell proliferation and promotes early neuronal differentiation [12]. Lacking EZH2 in neural progenitors also results in neuronal migration defects [15]. However, whether specific deletion of EZH2 in post-mitotic neurons has any effects on neural development is currently unknown; they might have phenotypes distinct from deleting EZH2 in neural stem cells. For instance, conditionally knocking out liver kinase B1 (LKB1) in neural progenitors using *Emx1-Cre* leads to defects in axon formation [37], whereas knocking out LKB1 in post-mitotic neurons using *Neurod6-Cre* has no effect on axon formation but results in axon branching defects [25]. In this study, we used the *Neurod6-Cre* mouse line to generate conditional *Ezh2* knockout mice specifically in post-mitotic neurons. Our results showed that neuronal EZH2 functions to regulate multiple neuronal morphogenesis processes *in vivo* during development and results in impaired cognitive function in adult mice.

In mature neurons, a recent study has shown that EZH1/EZH2 and H3K27me3 function to protect neurons from degeneration [38]. Two previous studies have reported impaired neuronal migration when EZH2 is knocked out [14] or knocked down [15] in neural progenitors. To date, however, no study has investigated the specific *in vivo* roles of neuronal EZH2 in neural development, which might be more relevant to neurodevelopmental disorders. Our study demonstrated that deleting neuronal EZH2 resulted in more complex dendritic arborization and higher dendritic spine density *in vivo*, indicating that it acts to maintain proper dendritic arborization and dendritic spine formation during development. In contrast, our recent study showed that EZH2 is necessary for axon growth and regeneration [39]. These results suggest that EZH2 functions to support axon growth during neuronal morphogenesis and in the meantime suppresses excessive dendritic development. It is likely that EZH2 regulates neuronal morphogenesis *via* H3K27me3-mediated gene repression. Many genes have been shown to regulate dendritic development, such as growth factors, small Rho GTPases, and cytoskeletal proteins [40–42]. How the expression of these genes is coordinated is unclear. Our study provides a potential mechanism in which EZH2 coordinately regulates genes related to dendritic development. Indeed, RNA-seq comparing neurons from the control and *Ezh2*^Δ/Δ^ mice has revealed many genes regulated by neuronal EZH2, including *Pak3, Igf,* and *Bdnf*, all of which have been shown to regulate dendritic development [43–47]. We provided evidence that *Pak3* expression was repressed by EZH2 and blocking PAK3 function reversed the increased dendritic spine density induced by EZH2 deletion. The results provided proof-in-principle evidence that neuronal EZH2 acts through small GTPase to regulate dendritic development. Although the expression of a dominant negative PAK3 rescued the dendritic spine defects in neurons of the *Ezh2*^Δ/Δ^ mice, it is unlikely to correct the cognitive behavioral defects in adult mice. One potential reason is that, as an epigenetic modulator to modify histone methylation, EZH2 presumably regulates the transcription of many downstream genes, which act to control different aspects of neural development at different stages. Indeed, RNA-seq results revealed that in neurons of *Ezh2*^Δ/Δ^ mutant mice, there were many DEGs, all of which coordinate to contribute to the normal cognitive functions of adult mice. Future in-depth studies of genes regulated by neuronal EZH2 will help to identify novel genes and pathways regulating *in vivo* dendritic development, providing new insights into the underlying genetic mechanisms. In this study, we also used RNA-seq to compare gene expression in post-mitotic neurons from wild-type and *Nestin-Cre*; *Ezh2*^f/f^ mice, in which *Ezh2* was knocked out in neural progenitors. The identified genes partially overlap with those obtained using neurons from the *Ezh2*^Δ/Δ^ mice, indicating that EZH2 at different developmental stages might regulate different sets of gene expression. In support, a recent study [31] has shown that *Ezh2* expression is positively regulated by *Chd8*, a top ASD-linked gene, in neural progenitors. In that study, knocking down *Chd8* resulted in the down-regulation of EZH2. However, upper-layer cortical neurons with reduced CHD8 and EZH2 proteins show decreased complexity of dendritic arborization, which is opposite to our results. Of course, we cannot rule out the possibility that CHD8 could regulate dendritic development independent of EZH2.

Dendritic arborization and dendritic spine formation are essential processes in the formation of functional circuits regulating cognitive function [48, 49]. In humans, mutations in the *Ezh2* gene underlie Weaver Syndrome, a genetic disease associated with intellectual disability. Both *Ezh2* and *Jmjd3* have been identified as ASD-associated genes [8, 9], and *Ezh2* is a downstream target gene of *Chd8* [31]. Our study revealed that specific deletion of *Ezh2* in early post-mitotic neurons resulted in impaired learning and memory defects in adult mice. It is worth noting that in our study there was no direct evidence supporting a causal relationship between the dendritic developmental defects and the cognitive defects found in adult mice. However, it is likely that changes in neuronal morphogenetic processes, especially the dendritic development induced by neuronal *Ezh2* deletion, might underlie some of the cognitive behavior phenotypes in adult *Ezh2*^Δ/Δ^ mice or potentially in patients with Weaver Syndrome or ASD. Because EZH2 has also been shown to regulate neurogenesis from adult neural stem cells [13], which might also contribute to the cognitive defects in adult *Ezh2*^Δ/Δ^ mice. Because we deleted EZH2 specifically in early post-mitotic neurons, the undifferentiated remaining neural stem cells presumably still express EZH2. Moreover, the origin of adult neural stem cells is currently not very clear. We thus do not know if the adult neural stem cells in *Ezh2*^Δ/Δ^ mice express EZH2 or not. Future detailed experiments are necessary to address this issue.

It seems confusing that the increased dendritic growth and spine density observed in our study was correlated with impaired learning and memory. We think that an optimal level of dendritic morphology and spine density is necessary for normal cognitive function. In support, previous studies have shown that either increased or decreased spine density is associated with impaired memory behavior [50, 51]. In particular, one study showed that greater spine densities were most commonly reported in autism patients with lower levels of cognitive function [51]. Additional more targeted cognitive behavioral tests are needed to further assess the roles of neuronal EZH2 in the regulation of cognitive functions. Previous studies have shown the roles of other epigenetic regulators in regulating dendritic development and cognitive function, such as histone deacetylase 2 (HDAC2) [50]. Conversely, behavioral training for learning and memory in adult mice also changes the histone modifications. For example, contextual fear conditioning increases the levels of acetylation at H3 lysine 14 (H3K14), phosphorylation at H3 serine 10 (H3S10), and trimethylation at H3 lysine 4 (H3K4me3) in the hippocampus [52]. Behavioral training on the Morris water maze increases the acetylation at H4 lysine 12 (H4K12) and the pan-acetylation of H2B [53]. Collectively, our study provides evidence that histone modification by EZH2 in post-mitotic neurons functions to regulate neural development with cognitive consequences in adult mice.

### Supplementary Information

Below is the link to the electronic supplementary material.

Supplementary file1 (PDF 1504 kb)

Supplementary file2 (XLSX 1125 kb)
