# Supplementary material for: Neuronal Histone Methyltransferase EZH2 Regulates Neuronal Morphogenesis, Synaptic Plasticity, and Cognitive Behavior in Mice
Source: Neurosci Bull. 2023 Jun 16;39(10):1512–32. doi: 10.1007/s12264-023-01074-1 (PMC10533778; doi:10.1007/s12264-023-01074-1)
Supplement: Supplementary file 1 — Supplementary file1 (PDF 1504 kb) [file 12264_2023_1074_MOESM1_ESM.pdf]

Supplementary Materials

Supplementary Figures

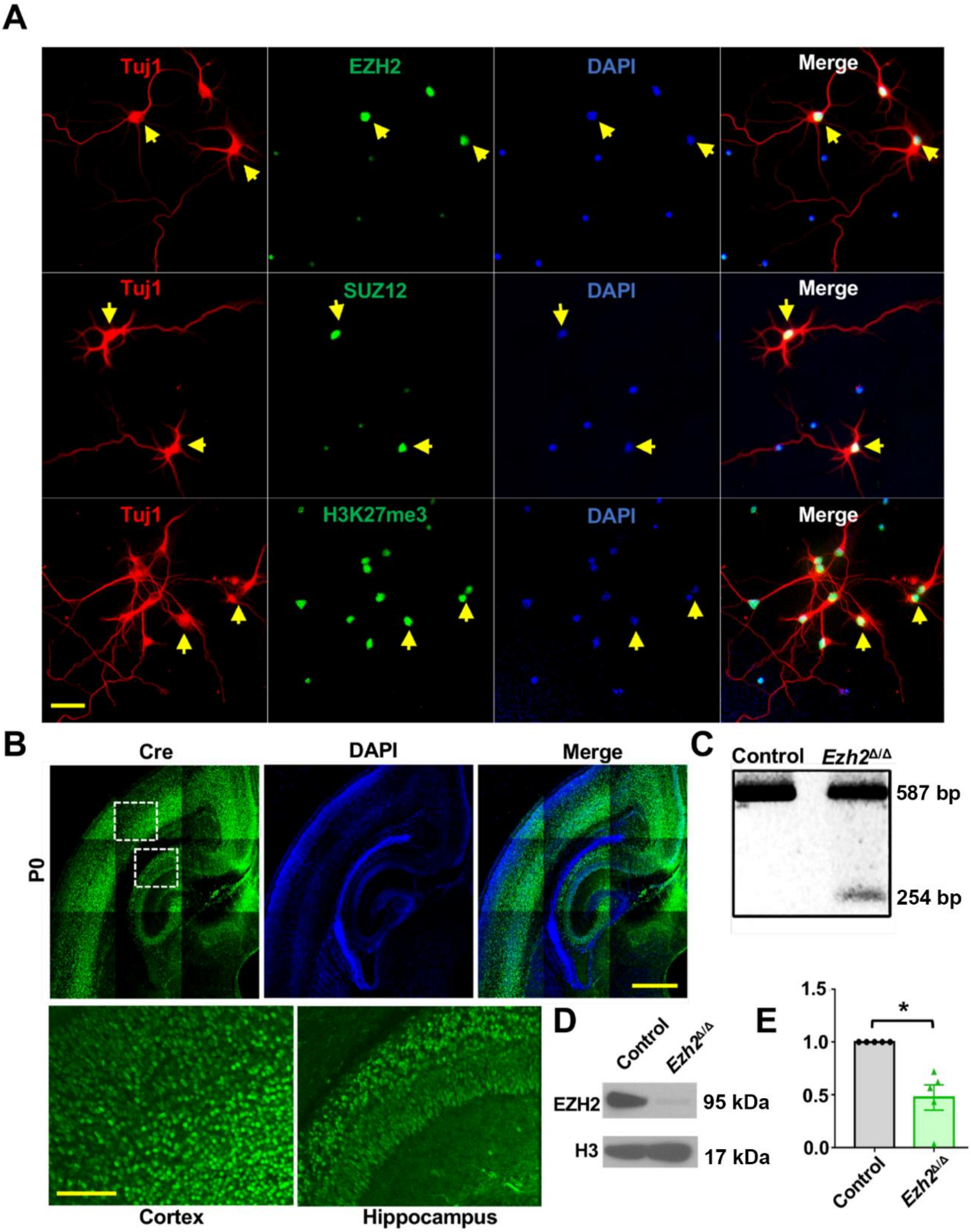

Fig. S1 Expression of EZH2, SUZ12, and H3K27me3 in cultured excitatory neurons. **A** Representative images showing the expression of EZH2, SUZ1, and H3K27me3 in cultured primary cortical neurons. Yellow arrowheads indicate Tuj1-positive neurons. Scale bar, 50 μm. **B** Upper: representative images of a P0 mouse coronal brain section stained with anti-Cre antibody showing the expression of Cre in the cortical and hippocampal regions. Lower: enlarged images of the two white dashed boxes in the upper panel. Scale bar, 500 μm (lower) and 2 mm (upper). **C** NeuroD6-Cre mediated recombination in the adult mouse brain is confirmed by RT-PCR. Representative PCR image showing a 254-bp fragment band generated by NeuroD6-Cre-mediated *Ezh2* deletion in *Neurod6-Cre*/*Ezh2*$^{f/+}$ heterozygous mice. The 587-bp band is the remaining wild-type EZH2 band. **D** Representative Western blot image (left) and quantification (right) showing the reduction of EZH2 by measuring the ratio of EZH2 and H3. The data are normalized to the control (P = 0.0117, n = 5 mice for each condition, unpaired Student's t-test). Data are represented as the mean ± SEM. *P <0.05, compared to control if not designated.

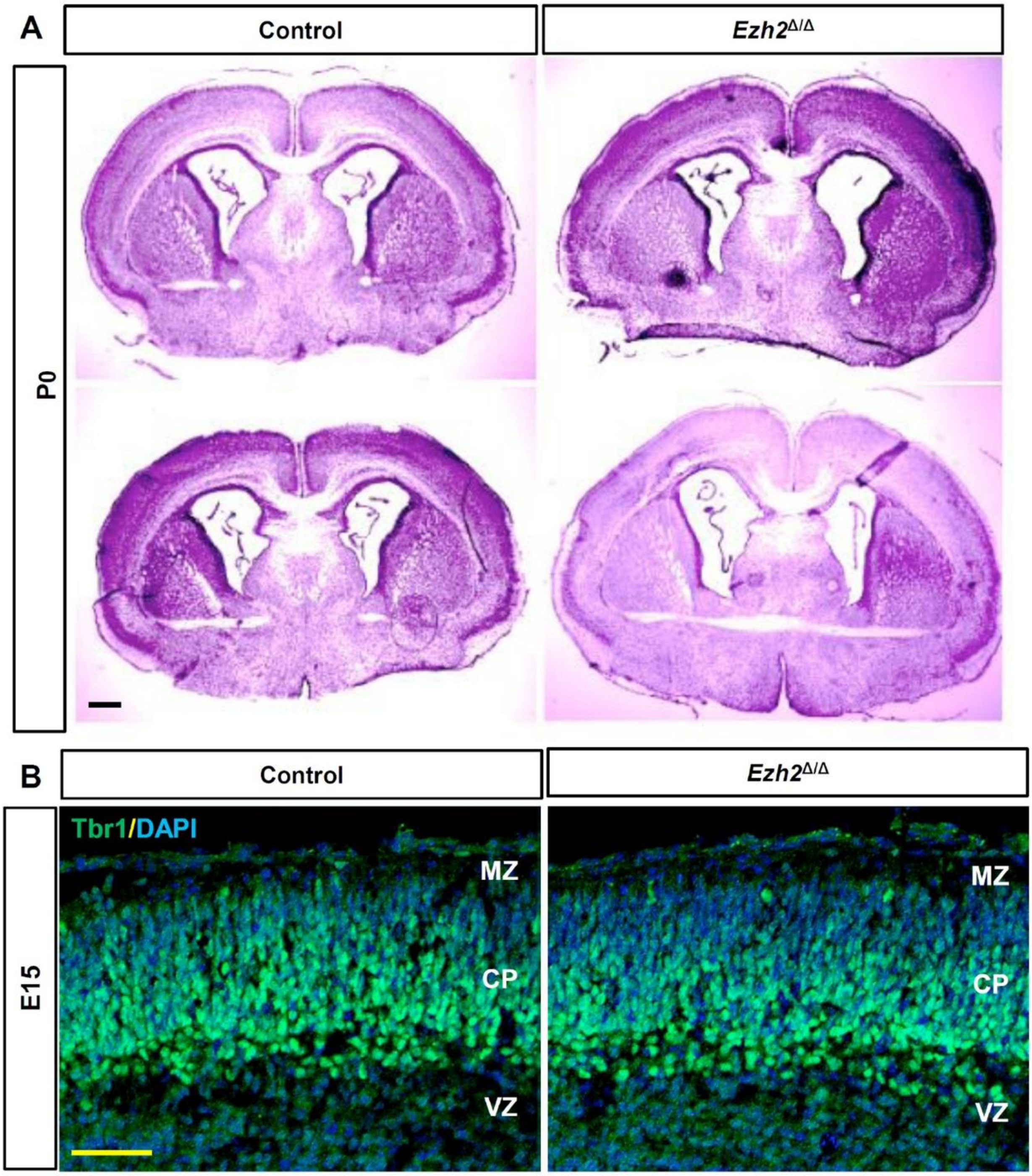

Fig. S2 Deletion of EZH2 manifests no structural brain abnormalities. **A** Representative Nissl staining images of coronal sections from P0 control and *Ezh2*$^{\Delta/\Delta}$ mice showing no obvious difference between the control and the *Ezh2*$^{\Delta/\Delta}$ mice. **B** Representative images of coronal brain sections stained with anti-Tbr1 antibody and DAPI showing that *Ezh2*$^{\Delta/\Delta}$ mice have normal early neurogenesis at embryonic day 14.5 (E14.5). Scale bars, 100 μm for both **A** and **B**. MZ, marginal zone; CP, cortical plate; VZ, ventricular zone.

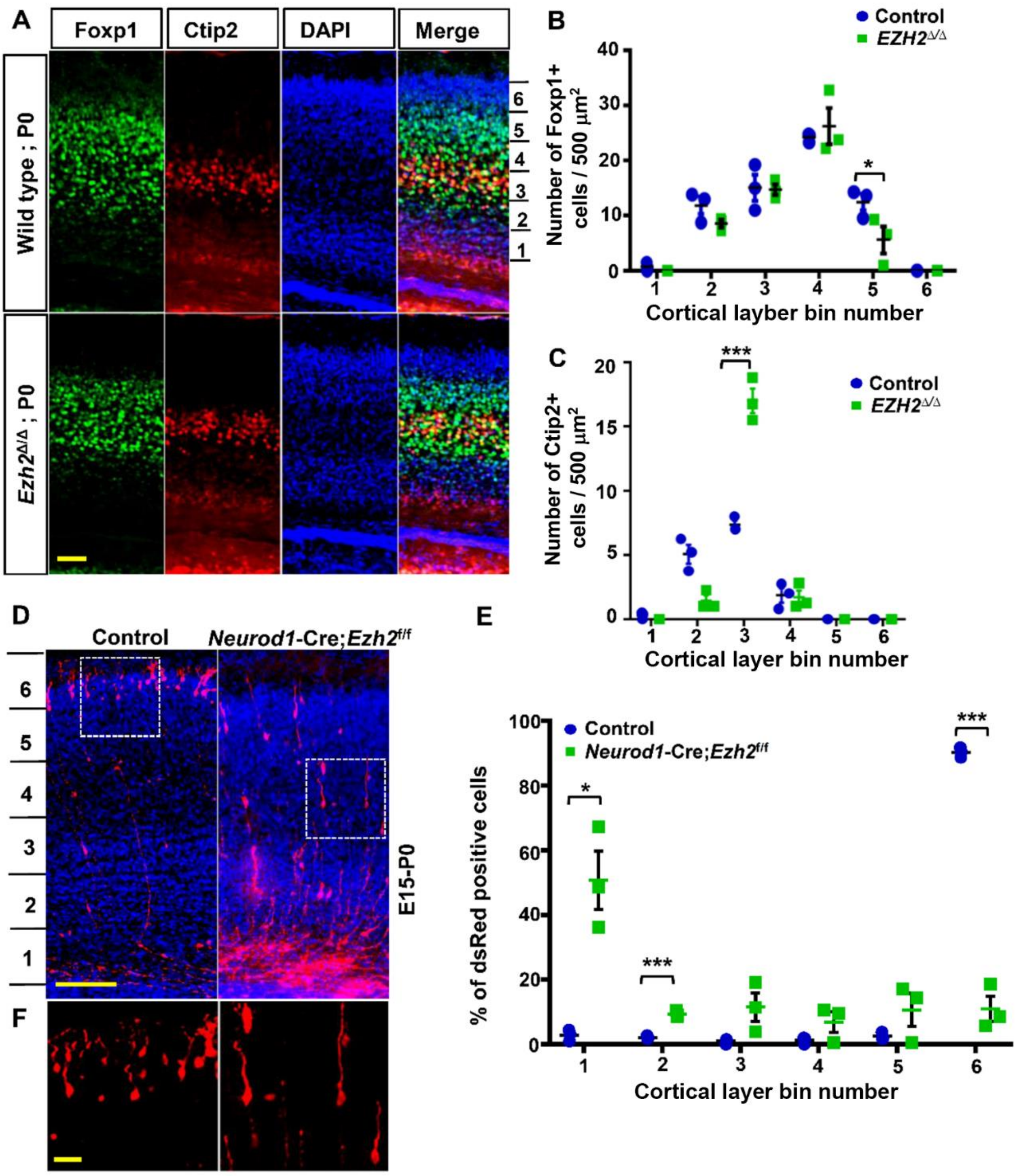

Fig. S3 Loss of EZH2 in cortical neurons delays the migration of upper cortical neurons. **A** Representative images of cortex immunostained for Foxp1 and Ctip2 in control and *Ezh2*$^{\Delta/\Delta}$ mice at P0. Foxp1 and Ctip2 labeling help to define layers II–IV and IV–V of the cortical plate, respectively. The distributions of labeled cells are assigned into 6 bins across the apicobasal axis. Scale bar, 100 μm. **B** Quantification of Foxp1-positive cells in 6 equal bins showing significantly reduced Foxp1-

positive neurons in Bin 5 (Bin 1, P = 0.2495; Bin 2, P = 0.1263; Bin 3, P = 0.9192; Bin 4, P = 0.5790; Bin 5, P = 0.0213, n = 3 mice for each condition, unpaired Student's *t*-test). **C** Quantification of Ctip2-positive cells in 6 equal bins showing they are significantly increased in Bin 3 (Bin 1, P = 0.1885; Bin 2, P = 0.0822; Bin 3, P = 0.016; Bin 4, P = 0.8954, n = 3 mice for each condition, unpaired Student's *t*-test). **D** Representative confocal images of $Ezh2^{f/f}$ mouse cortices *in utero* electroporated with dsRed or dsRed/*Neurod1-Cre*. The electroporation was done at E15, and pups were sacrificed at P0 for analysis. The two white dashed boxes in the upper panels are enlarged and presented in the lower panels. Scale bars, 200 μm in the upper panels and 50 μm in the lower panels. **E** Quantification of dsRed-positive cells in mouse cortices as in **D** showing a significant increase in the number of cells in Bin 1 but a reduced number of cells in Bin 6 in mice electroporated with *Neurod1-Cre* (Bin 1, P = 0.0325; Bin 2, P = 0.0019; Bin 3, P = 0.1375; Bin 4, P = 0.2140; Bin 5, P = 0.2502; Bin 6, P = 0.0016; n = 3 mice for each condition, unpaired Student's *t*-test). Data are presented as the mean ± SEM. *P <0.05, **P <0.01, ***P <0.001, compared to control if not designated.

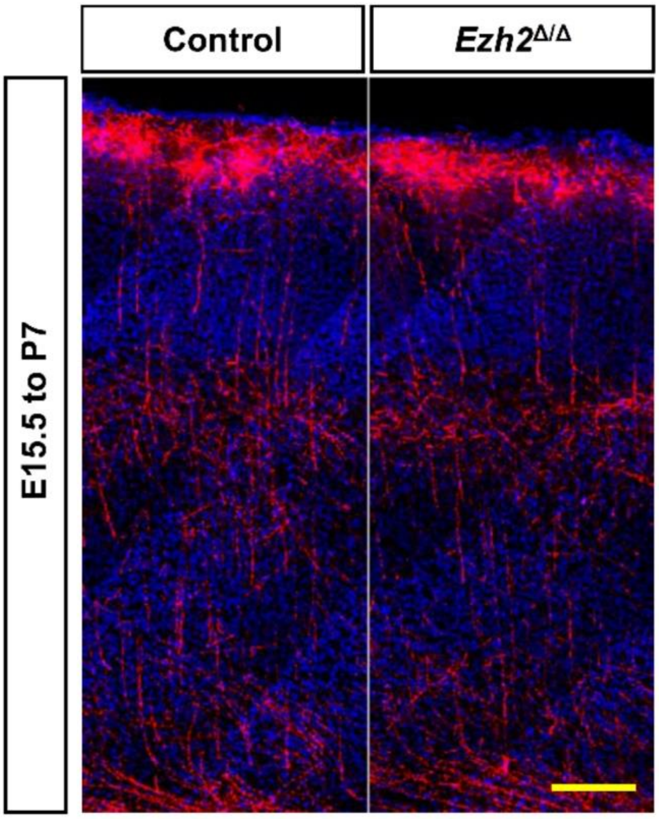

Fig. S4 Lack of EZH2 has little effect on cortical neuronal migration at P7. Representative confocal images of mouse cortices *in utero* electroporated with dsRED or dsRED/Neurod1-Cre. The electroporation was performed at E15 and the pups were harvested at P7. Scale bar, 200 μm.

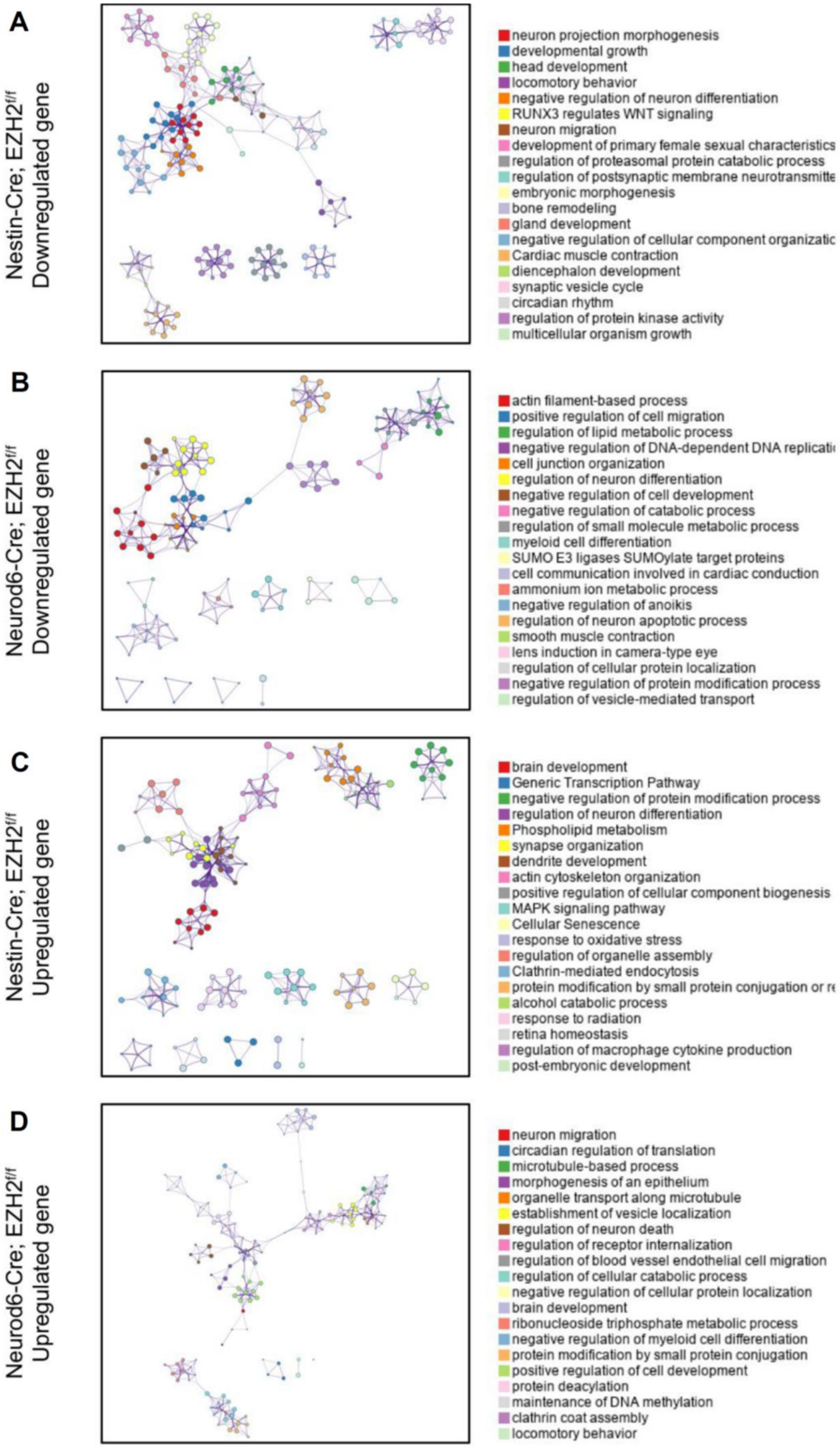
A
Nestin-Cre; EZH2f/f
Downregulated gene
neuron projection morphogenesis
developmental growth
head development
locomotory behavior
negative regulation of neuron differentiation
RUNX3 regulates WNT signaling
neuron migration
development of primary female sexual characteristics
regulation of proteasomal protein catabolic process
regulation of postsynaptic membrane neurotransmitte
embryonic morphogenesis
bone remodeling
gland development
negative regulation of cellular component organizatic
Cardiac muscle contraction
diencephalon development
synaptic vesicle cycle
circadian rhythm
regulation of protein kinase activity
multicellular organism growth
B
Neurod6-Cre; EZH2f/f
Downregulated gene
actin filament-based process
positive regulation of cell migration
regulation of lipid metabolic process
negative regulation of DNA-dependent DNA replicati
cell junction organization
regulation of neuron differentiation
negative regulation of cell development
negative regulation of catabolic process
regulation of small molecule metabolic process
myeloid cell differentiation
SUMO E3 ligases SUMOylate target proteins
cell communication involved in cardiac conduction
ammonium ion metabolic process
negative regulation of anoikis
regulation of neuron apoptotic process
smooth muscle contraction
lens induction in camera-type eye
regulation of cellular protein localization
negative regulation of protein modification process
regulation of vesicle-mediated transport
C
Nestin-Cre; EZH2f/f
Upregulated gene
brain development
Generic Transcription Pathway
negative regulation of protein modification process
regulation of neuron differentiation
Phospholipid metabolism
synapse organization
dendrite development
actin cytoskeleton organization
positive regulation of cellular component biogenesis
MAPK signaling pathway
Cellular Senescence
response to oxidative stress
regulation of organelle assembly
Clathrin-mediated endocytosis
protein modification by small protein conjugation or re
alcohol catabolic process
response to radiation
retina homeostasis
regulation of macrophage cytokine production
post-embryonic development
D
Neurod6-Cre; EZH2f/f
Upregulated gene
neuron migration
circadian regulation of translation
microtubule-based process
morphogenesis of an epithelium
organelle transport along microtubule
establishment of vesicle localization
regulation of neuron death
regulation of receptor internalization
regulation of blood vessel endothelial cell migration
regulation of cellular catabolic process
negative regulation of cellular protein localization
brain development
ribonucleoside triphosphate metabolic process
negative regulation of myeloid cell differentiation
protein modification by small protein conjugation
positive regulation of cell development
protein deacylation
maintenance of DNA methylation
clathrin coat assembly
locomotory behavior

Fig. S5 Network of enriched GO terms among each differentially-expressed gene set depicts connections among GO terms. Subgroups of the enriched GO terms shown in Fig. 6 have been plotted and visualized using Metascape (http://metascape.org) and Cytoscape5. The program constructs a GO term network among terms with a similarity >0.3, which are connected by edges. Each node represents one enriched GO term, where node size is the number of genes within a GO term. GO terms within the same cluster are visualized with the same color. *P*-values of the enrichments refer back to Fig. 6, which shows the maximal *P*-value is 1.0×10^{-3}. **A** GO term network of down-regulated genes in the *Nestin-Cre*;*Ezh2*$^{f/f}$ compared with the control. **B** GO term network of down-regulated genes in the *Neurod6 (Nex)-Cre;Ezh2*$^{f/f}$ compared with the control. **C** GO term network of up-regulated genes in the *Nestin-Cre; Ezh2*$^{f/f}$ compared with the control. **D** GO term network of up-regulated genes in the *Neurod6 (Nex)-Cre*;*Ezh2*$^{f/f}$ compared with the control.

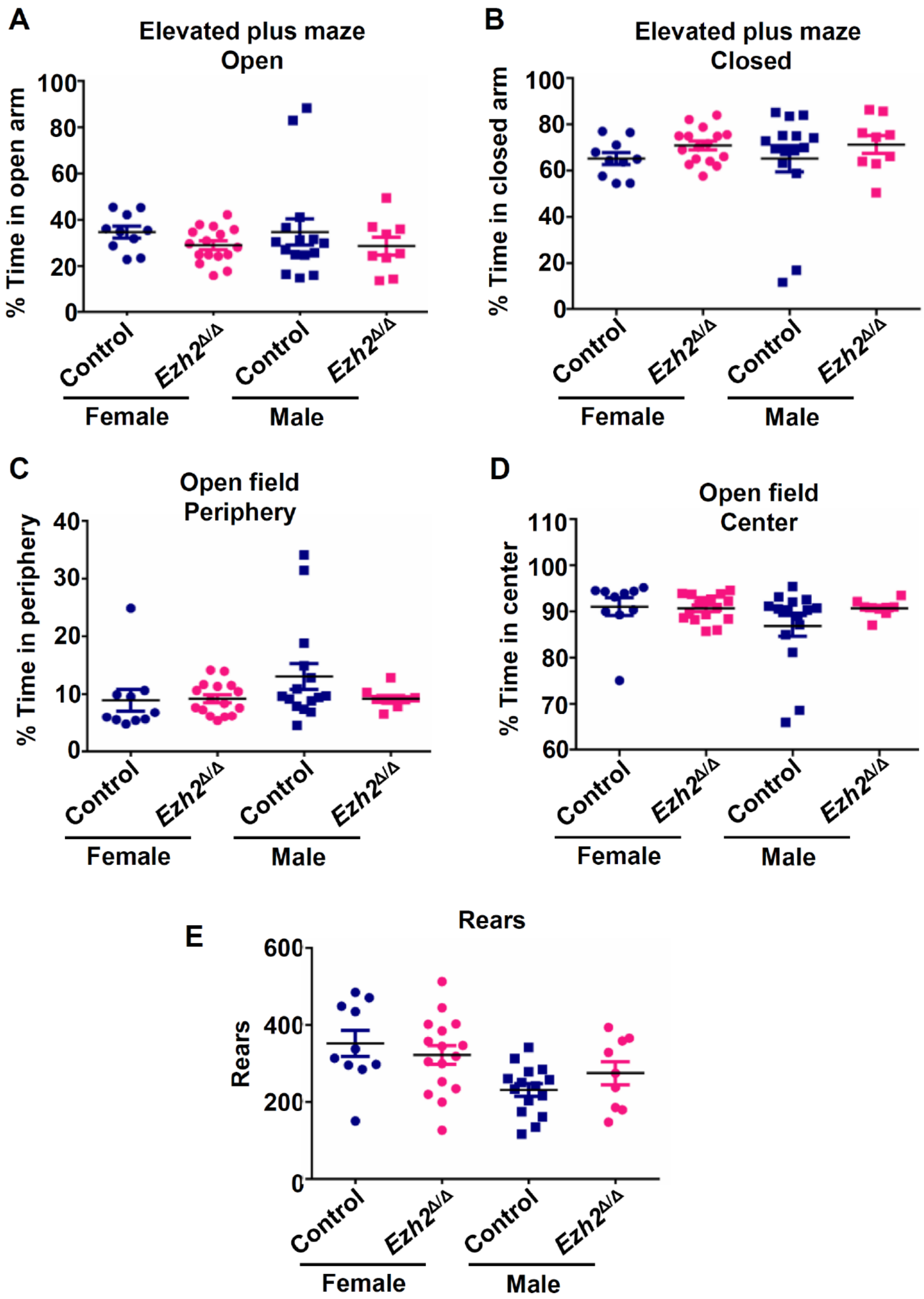

A
Elevated plus maze
Open
% Time in open arm
100
80
60
40
20
0
Control
Ezh2Δ/Δ
Control
Ezh2Δ/Δ
Female
Male
B
Elevated plus maze
Closed
% Time in closed arm
100
80
60
40
20
0
Control
Ezh2Δ/Δ
Control
Ezh2Δ/Δ
Female
Male
C
Open field
Periphery
% Time in periphery
40
30
20
10
0
Control
Ezh2Δ/Δ
Control
Ezh2Δ/Δ
Female
Male
D
Open field
Center
% Time in center
110
100
90
80
70
60
Control
Ezh2Δ/Δ
Control
Ezh2Δ/Δ
Female
Male
E
Rears
Rears
600
400
200
0
Control
Ezh2Δ/Δ
Control
Ezh2Δ/Δ
Female
Male

Fig. S6 *Ezh2*$^{\Delta/\Delta}$ mutant mice show little defects in the elevated plus maze test and the open field test. **A** Quantification of time spent in the open arms of the elevated plus maze (female: n = 10 mice for control mice and n = 16 for *Ezh2*$^{\Delta/\Delta}$ mice, P = 0.6808; male: n = 15 for control mice and n = 9 for *Ezh2*$^{\Delta/\Delta}$ mice, P = 0.6093, unpaired Student's t-test). **B** Quantification of time spent in the closed arms of the elevated plus maze (female: n = 10 for the control group and n = 16 for the *Ezh2*$^{\Delta/\Delta}$ group, P = 0.6808; male: n = 15 for control mice and n = 9 for *Ezh2*$^{\Delta/\Delta}$ mice, P = 0.6093, unpaired Student's t-test). **C–E** Quantification of alterations in time spent in the peripheral zone (**C**), central zone (**D**), and rearing behavior (**E**). Female: n = 10 for control mice and n = 16 for *Ezh2*$^{\Delta/\Delta}$ mice; male: n = 15 for control mice and n = 9 for *Ezh2*$^{\Delta/\Delta}$ mice. Open field peripheral: P = 0.8551 for female mice and P = 0.2052 for male mice, unpaired Student's t-test. Open field center: P = 0.8561 for female mice and P = 0.3643 for male mice, unpaired Student's t-test. Open field rearing: P = 0.4703 for female mice and P = 0.1854 for male mice, unpaired Student's t-test. Data are presented as the mean ± SEM. n.s., no significant difference, compared to control if not designated.
